# Characterization of Rookie Season Injury and Illness and Career Longevity Among National Basketball Association Players

**DOI:** 10.1001/jamanetworkopen.2021.28199

**Published:** 2021-10-04

**Authors:** Chelsea L. Martin, Amelia J. H. Arundale, Stefan Kluzek, Tyler Ferguson, Gary S. Collins, Garrett S. Bullock

**Affiliations:** 1 ATI Physical Therapy, Greenville, South Carolina; 2 Icahn School of Medicine at Mount Sinai Health System, New York, New York; 3 Red Bull Athlete Performance Center, Red Bull GmBH, Thalgau, Austria; 4 Centre for Sport, Exercise and Osteoarthritis Research Versus Arthritis, University of Oxford, United Kingdom; 5 Nuffield Department of Orthopaedics, Rheumatology, and Musculoskeletal Sciences, University of Oxford, Oxford, United Kingdom; 6 University of Nottingham, Nottingham, United Kingdom; 7 Big Data Institute, University of Oxford, United Kingdom; 8 Centre for Statistics in Medicine, Nuffield Department of Orthopaedics, Rheumatology, and Musculoskeletal Sciences, University of Oxford, Oxford, United Kingdom; 9 Oxford University Hospitals NHS Foundation Trust, Oxford, United Kingdom; 10 Department of Orthopaedic Surgery, Wake Forest School of Medicine, Winston-Salem, North Carolina

## Abstract

**Importance:**

There is limited research investigating injury and illness among professional basketball players during their rookie season. By improving the understanding of injury incidence and risk specific to rookie players, sports medicine clinicians may be able to further individualize injury mitigation programs that address the unique needs of rookie players.

**Objective:**

To compare incidence and rate ratio (RR) of injury and illness among professional National Basketball Association (NBA) players in their rookie season with veteran players and to explore the association of sustaining an injury rookie season with career longevity.

**Design, Setting, and Participants:**

This retrospective cohort study used an online data repository and extracted publicly available data about NBA players between the 2007 and 2008 season to the 2018 and 2019 season. Available data for initial injury and all subsequent injuries were extracted during this time frame.

**Exposures:**

Injury and illness based on injury status during the rookie season of professional NBA players.

**Main Outcomes and Measures:**

Injury and illness incidence and RR. Association of injury during the rookie season with career longevity was assessed via Poisson regressions.

**Results:**

Of the 12 basketball seasons analyzed, 904 NBA players were included (mean [SD] age, 24.6 [3.9] years; body mass index, 24.8 [1.8]). The injury and illness incidence for rookie players was 14.28 per 1000 athlete game exposures (AGEs). Among all body regions, ankle injuries had the greatest injury incidence among players injured during their rookie season (3.17 [95% CI, 3.15-3.19] per 1000 AGEs). Rookie athletes demonstrated higher RR compared with veterans across multiple regions of the body (ankle: 1.32; 95% CI, 1.12 to 1.52; foot/toe: 1.29; 95% CI, 0.97 to 1.61; shoulder/arm/elbow: 1.43; 95% CI, 1.10 to 1.77; head/neck: 1.21; 95% CI, 0.61 to 1.81; concussions: 2.39; 95% CI, 1.89 to 2.90; illness: 1.14; 95% CI, 0.87 to 1.40), and demonstrated a higher rate of initial injuries compared with veteran players (1.41; 95% CI, 1.29 to 1.53). Players who sustained an injury rookie season demonstrated an unadjusted decrease in total seasons played (–0.4 [95% CI, –0.5 to –0.3] log years; *P* < .001), but this decrease was not observed within adjusted analysis (0.1 [95% CI, –0.1 to 0.2] log years; *P* = .36).

**Conclusions and Relevance:**

In this study, rookie athletes demonstrated the highest injury incidence at the ankle and increased RR across multiple regions. These findings may reflect differences in preseason conditioning or load variables impacting rookie athletes and warrant further investigation. Future research is needed to determine the association of cumulative injury burden vs a singular injury event on career longevity.

## Key Points

**Question:**

What is the rate ratio (RR) of injury and illness among rookie and veteran professional basketball players, and is sustaining an injury during the rookie season associated with career longevity?

**Findings:**

In this cohort study of 904 professional basketball players, rookies demonstrated higher RRs of injury across multiple categories. Those who sustained an injury during their rookie season did not demonstrate a significant difference in total seasons played.

**Meaning:**

In this study, rookie athletes demonstrated unique RR profiles compared with veterans, but singular injury occurrence during rookie season was not associated with career longevity; these findings suggest a need to investigate cumulative injury burden and the association with career longevity.

## Teaser

This cohort study compares the incidence and rate ratio of injury and illness among rookie professional basketball players with veteran players and explores the association of sustaining an injury rookie season with career longevity.

## Introduction

The National Basketball Association (NBA) is the highest men’s professional basketball league in North America.^1^ Basketball is a sport requiring physical contact, jumping, and pivoting at high speeds.^1^ Understandably, injury incidence is high across multiple body regions^2^ with time loss injury cost estimated at $300 million each season.^1^ Furthermore, time loss injuries may be associated with an athlete’s performance upon return to play.^3,4^ Because of the financial and physical burden associated with NBA injuries, basketball stakeholders, including coaches, owners, players, and clinicians, have sought to mitigate injuries.^5^ A systematic approach in injury prevention research has been suggested by van Mechelen et al,^6^ beginning with injury surveillance to increase understanding of incidence and severity.

NBA rookies are athletes who are competing in their first year in the professional league. Since 2006, players are required to be a minimum age of 19 years to be eligible for the NBA draft; as a result, more collegiate players have opted into the NBA draft after only 1 collegiate season, decreasing the mean draft age.^7^ With younger players being drafted, rookie players may present with less physical maturation and have difficulty managing higher training loads and increased games per season.^8,9,10^ This difficulty in load management may reflect decreased fitness and development. Although injury incidence research among NBA rookies has been sparse, previous research among military recruits^11^ and National Football League combine participants^12^ indicates that previous injury and lower fitness levels indicate future injury risk and decreased career longevity. Therefore, identifying injury incidence and risk specific to rookies is necessary to individualize injury mitigation programs that address the unique needs of rookie players.

Publicly available data provide information needed to determine time loss injury incidence among rookie and veteran players.^13,14^ Transparent and reproducible automated methods of extracted publicly available data facilitate collaborations and data distribution among organizations and stakeholders.^15^ This method was used to study NBA players for all injury and illness incidence during the 1999 to 2000 season, and previous reports demonstrated high reporting reliability.^13^ Technological advances and public reporting of injury data have improved since this public data NBA injury study, improving the precision of injury and illness results.^16^ Furthermore, the use of computer iterative repeatable methods allows for an efficient method of data extraction and repeatability and offers the potential for shared leaguewide injury risk identification and injury mitigation programs.^17^ This study used publicly available data to compare incidence and risk of injury and illness among rookie professional basketball players with veteran players and determine whether a player sustaining an injury rookie season was associated with career longevity.

## Methods

### Study Design

This cohort study used publicly available data about NBA players for this investigation through a computer iterative reproducible method.^17^ Three online resources were accessed to create a combined data set for this study (eAppendix 1 in the Supplement).^18,19,20^ NBA stakeholders were included for expertise in research question development and clinical interpretability. Informed consent and institutional review board approval was waived because data were publicly available and deidentified. To ensure transparency of research findings, the Strengthening the Reporting of Observational Studies in Epidemiology (STROBE) reporting guideline was used.

### Participants

Participants were NBA players who were 18 years or older and had competed in at least 1 professional season between 2008 and 2019. Rookie players were defined as players competing in their first season of the respective year analyzed, whereas all other players competing during the same year were classified as veterans. Age and body mass index (BMI), calculated as weight in kilograms divided by height in meters squared, was extracted through the aforementioned online resources and recorded based on season the injury occurred. Career longevity was defined as the total number of seasons played by athletes. Draft order among participants were classified as early (ie, 1-20 draft pick), middle (ie, 21-40 draft pick), or late (ie, 41-60 draft pick).

### Injury and Illness Classification

Time loss injury was defined as an injury to a tendon, ligament, nerve, muscle, or bone followed by at least 1 day of missed practice or competition.^21^ An illness was defined as a complaint or disorder reported by a player and his team, not related to injury, resulting in at least 1 day of missed practice or competition.^2,22^ Season time frame included only regular season games. Injuries were categorized by body part,^23^ and severity was calculated as games missed because of injury or illness.^24^

The injury or illness date resolution was based on the first game a player competed in after time loss injury. Therefore, the date of injury or illness data were combined with game data to determine the first game played by each player after an incident reported. Two counts were also added as a measure of injury severity: the number of days an injury lasted, and the number of games missed during the injury. Injury severity was further stratified into injury groups defined as slight (1 missed game), minor (2-3 missed games), moderate (4-13 missed games), or severe (≥14 missed games).^24^

### Data Extraction

A data repository was accessed on December 1, 2019, and data were retrieved from inception to December 1, 2019.^18^ The 2019 to 2020 season was excluded from analyses because of the COVID-19 pandemic shutdown, and data were found to be inconsistent before the 2007 to 2008 seasons; therefore, data between 2007 to 2008 and 2018 to 2019 season were included for analyses. Injury data scraping was performed in R version 4.02, using the rvest, tm, and xm12 packages (R Project for Statistical Computing). Data retrieved for all seasons and players in the study period included injury during game or practice, player position, games played, games missed, and season summary statistics. The custom R package NBAinjuries was used to extract these data. Further information on data scraping methodology, code, and raw data obtained are provided in eAppendix 1 and eAppendix 2 in the Supplement.

### Data Reduction

Inconsistencies were observed in the data before the 2007 to 2008 season. Thus, data before the 2007 to 2008 season were removed from the data set. The data source contained reliable data of injury occurrence but frequently did not report the data of injury resolution.^18^ Automated internal validation checks were performed to ensure that improbable values did not occur as part of this data preprocessing sample.

### External Validation

Data external validation was performed by 2 external examiners (graduate students at the University of Nottingham) using a number generator that randomly selected 180 data points from the data set. The primary investigator trained the 2 external examiners in methods to assess the validity of injury occurrence and body part from publicly sourced internet websites (ESPN.com, NBA.net, or pertinent team websites) and compared findings with the randomly selected data set. The randomly selected data demonstrated a low positive predictive value of 44.7% for the exact injury date but a strong predictive value for missed games (84.1%) and injury site (95.6%). The low positive predictive value observed for the date of injury was because the data set displayed the first game the athlete missed because of injury, whereas the validation found the actual date the injury occurred. Furthermore, the discrepancies observed for missed games was because athletes were included on the roster but did not participate in the NBA game. Such missed game discrepancies likely represented an athlete cleared to play but unable to play to their full potential because of their injury or illness and thus not selected by the coach to play.

### Statistical Analysis

Descriptive data were described as mean (SD), median (IQR), or count (percentage). Injury and illness incidence were calculated by the sum of injuries and divided by the sum of player-games, multiplied by 1000 times athlete game exposures (AGEs). Because both practice and game injuries were reported but missed games demonstrated a higher predictive value over injury date, the sum of player-game exposures was used to determine an appropriate exposure measurement. Injury incidence was reported based on when the injury occurred (ie, during rookie season or subsequent seasons) and stratified by body part and injury severity. Rate ratio (RR) of injury among rookie players was calculated by the incidence of rookie players divided by incidence of veteran players and stratified by body part and initial vs subsequent injury. Because injury incidents followed assumptions of a Poisson distribution, Poisson regression models were performed to compare the association of injury incurred during athletes’ rookie seasons with career longevity (ie, seasons postinjury). Illness was excluded from these analyses. Confounders were adjusted for continuous variables in the models and included age, BMI, position, year of injury, the severity of injury, and overall draft pick number. Sensitivity analyses were performed to analyze the association of injury during an athlete’s rookie season with career longevity for severe and lower extremity injuries. All analyses were performed in R version 4.02 (R Project for Statistical Computing) with the cleaning and descriptive analyses performed with the dplyr package and Poisson models performed with the stats package. Rookie and veteran player characteristics were compared via 2-tailed *t* tests and, and statistical significance was set at *P* < .05. Data were collected on December 1, 2019, and analyzed between July 2020 to August 2020.

## Results

In this cohort study, 904 players were recorded for the 2007 to 2008 through 2018 to 2019 NBA seasons (mean [SD] age, 24.6 [3.9]; mean [SD] BMI, 24.8 [1.8]) (Table 1). In total, 5364 injuries or illnesses among 830 658 player-games were recorded from 2007 to 2008 through 2018 to 2019 seasons. The incidence of overall combined injuries and illnesses among players who sustained an injury during their rookie season was 14.28 per 1000 AGEs compared with players who did not sustain an injury or illness during their rookie season (13.79 per 1000 AGE’s). Players who sustained an injury during their rookie season played a mean (SD) of 4.9 (2.9) seasons and had a median draft pick number of 17 (IQR, 6-30) (Table 1). Players who did not experience an injury during their rookie season played a mean (SD) of 7.6 (3.4) seasons and had a median draft pick of 16 (IQR, 7-27) (Table 1).

**Table 1.** Participant Characteristics^a^

Variable	All injured NBA players (N = 904)	Sustained an injury rookie season (N = 178)	Did not sustain an injury during rookie season (n = 726)	*P* value
BMI, mean (SD)	24.8 (1.8)	24.8 (1.3)	25.0 (1.7)	.32
Seasons played, mean (SD)	5.0 (3.2)	4.9 (2.9)	7.6 (3.4)	.73
Age, mean (SD), y	24.6 (3.9)	22.8 (2.1)	25.0 (4.1)	.43
Draft order, median (IQR)	16 (7-28)	17 (6-30)	16 (7-27)	.09
Position, No. (%)				
Center	185 (20.5)	65 (21.3)	120 (20.0)	.35
Forward	357 (39.5)	125 (41.0)	232 (38.7)	.20
Guard	362 (40)	115 (37.7)	247 (41.2)	.15
Injury incidence (per 1000 AGE)	13.82	14.28	13.79	.91
Injury severity, median (IQR)^b^	3 (1-7)	3 (1-8)	3 (1-7)	.06

Abbreviations: AGE, athlete game exposure; BMI, body mass index (calculated as weight in kilograms divided by height in meters squared).

^a^ Characteristics were compared between players who did or did not sustain an injury during their rookie season.

^b^ Injury severity was determined by the number of games missed.

### Injury Incidence and RR by Location and Severity

The greatest injury and illness incidence among players who sustained an injury during their rookie season was to the ankle (3.17; 95% CI, 3.15-3.19 per 1000 AGEs) (Table 2). Rookie athletes demonstrated a higher RR of injury to the ankle (1.32; 95% CI, 1.12 to 1.52), foot and/or toe (1.29; 95% CI, 0.97 to 1.61), shoulder, arm, and/or elbow (1.43; 95% CI, 1.10 to 1.77), and head and/or neck (1.21; 95% CI, 0.61 to 1.81) compared with veteran players. Furthermore, higher RR of concussions (2.39; 95% CI, 1.89 to 2.90) and illness (1.14; 95% CI, 0.87 to 1.40) among rookie athletes were found (Table 3). Rookie athletes demonstrated a higher rate of initial injuries compared with veteran players (1.41; 95% CI, 1.29 to 1.53) (eTable 8 in the Supplement). The greatest injury and illness incidence among those who did not sustain an injury rookie season was to the knee (2.52; 95% CI, 2.46-2.58) (Table 2). Notably, nonrookie (veteran) athletes demonstrated a 47% increased rate of trunk, back, and/or buttock injuries compared with rookie players (Table 3). Regarding injury severity, players who sustained an injury during their rookie season had a higher incidence of severe injuries to the ankle; groin, hip, and/or thigh; shoulder, arm, and/or elbow, or shoulder/arm/elbow compared with players who did not sustain an injury during their rookie season (ankle: 0.44 [95% CI, 0.43-0.44] per 1000 AGEs vs 0.24 [95% CI, 0.22-0.26] per 1000 AGEs; groin, hip, and/or thigh: 0.32 per 1000 AGEs vs 0.23 per 1000 AGEs; shoulder, arm, and/or elbow, 0.44 [95% CI, 0.43-0.44] per 1000 AGEs vs 0.13 [95% CI, 0.12-0.15] per 1000s AGEs) (Table 2). Additional information regarding injury severity comparisons for overall and temporal trends among veteran and rookie players can be found in eTable 1 to eTable 4 in the Supplement.

**Table 2.** Injury and Illness Incidence by Location and Severity

Body part or illness	Injury count, No.	Games missed, median (IQR), No.^**a**^	Injuries, incidence (95% CI), per 1000 AGEs^**b**^	Injury severity incidence, (95% CI), per 1000 AGEs			
				Slight	Minor	Moderate	Severe
Ankle							
All injured NBA players	957	3 (1-6)	2.47 (2.40-2.53)	0.78 (0.74-0.81)	0.70 (0.67-0.73)	0.73 (0.70-0.76)	0.26 (0.24-0.28)
Injury occurred rookie season	109	3 (1-6)	3.17 (3.15-3.19)	0.81 (0.80-0.82)	1.08 (1.06-1.09)	0.84 (0.83-0.85)	0.44 (0.43-0.44)
Injury occurred nonrookie season	848	3 (1-6)	2.40 (2.34-2.45)	0.78 (0.74-0.81)	0.66 (0.63-0.69)	0.72 (0.69-0.75)	0.24 (0.22-0.26)
Knee							
All Injured NBA players	974	3 (1-12)	2.51 (2.45-2.57)	0.71 (0.67-0.74)	0.62 (0.59-0.65)	0.60 (0.57-063)	0.59 (0.56-0.61)
Injury occurred rookie season	83	3 (1-12)	2.41 (2.40-2.43)	0.81 (0.80-0.82)	0.49 (0.49-0.50)	0.55 (0.54-0.56)	0.55 (0.54-0.56)
Injury occurred nonrookie season	891	3 (1-12)	2.52 (2.46-2.58)	0.70 (0.66-0.73)	0.63 (0.60-0.66)	0.61 (0.58-0.63)	0.59 (0.56-0.62)
Groin/hip/thigh							
All injured NBA players	765	3 (1-7)	1.97 (1.92-2.02)	0.54 (0.51-0.57)	0.57 (0.54-0.60)	0.62 (0.59-0.65)	0.23 (0.22-0.25)
Injury occurred rookie season	54	4 (1-6)	1.57 (1.56-1.58)	0.44 (0.43-0.44)	0.32 (0.31-0.33)	0.49 (0.49-0.50)	0.32 (0.31-0.33)
Injury occurred nonrookie season	711	3 (1-6)	2.01 (1.95-2.06)	0.55 (0.52-0.58)	0.60 (0.57-0.62)	0.64 (0.60-0.67)	0.23 (0.21-0.24)
Illness							
All injured NBA players	600	1 (1-3)	1.55 (1.50-1.59)	0.83 (0.79-0.86)	0.39 (0.37-0.42)	0.26 (0.24-0.28)	0.06 (0.05-0.07)
Injury occurred rookie season	60	2 (1-3)	1.74 (1.73-1.76)	0.81 (0.80-0.82)	0.49 (0.49-0.50)	0.29 (0.28-0.30)	0.15 (0.14-0.15)
Injury occurred nonrookie season	540	1 (1-3)	1.53 (1.48-1.57)	0.83 (0.80-0.86)	0.38 (0.36-0.41)	0.26 (0.24-0.28)	0.05 (0.04-0.06)
Trunk, back, and/or buttocks							
All injured NBA players	566	2 (1-5)	1.46 (1.41-1.50)	0.57 (0.54-0.60)	0.42 (0.40-0.45)	0.31 (0.29-0.33)	0.14 (0.13-0.16)
Injury occurred rookie season	35	2 (1.5-5)	1.02 (1.01-1.03)	0.26 (0.26-0.27)	0.32 (0.31-0.33)	0.29 (0.29-0.30)	0.15 (0.14-0.15)
Injury occurred nonrookie season	531	2 (1-5)	1.50 (1.46-1.55)	0.60 (0.57-0.63)	0.43 (41-0.46)	0.31 (0.29-0.33)	0.16 (0.14-0.17)
Foot and/or toe							
All injured NBA players	385	3 (1-10)	0.99 (0.95-1.03)	0.26 (0.24-0.28)	0.24 (0.22-0.26)	0.28 (0.26-0.30)	0.21 (0.19-0.23)
Injury occurred rookie season	43	6 (1. 7.5)	1.25 (1.24-1.26)	0.29 (0.28-0.30)	0.20 (0.20-0.21)	0.38 (0.37-0.39)	0.38 (0.37-0.39)
Injury occurred nonrookie season	342	3 (1-10)	0.97 (0.93-1.00)	0.26 (0.24-0.28)	0.25 (0.23-0.26)	0.27 (0.24-0.29)	0.19 (0.18-0.21)
Forearm, wrist, and/or hand							
All injured NBA players	324	5 (2-15)	0.84 (0.80-0.87)	0.20 (0.19-0.22)	0.17 (0.15-0.18)	0.21 (0.19-0.23)	0.25 (0.23-0.27)
Injury occurred rookie season	27	9 (2.5-15)	0.78 (0.77-0.80)	0.17 (0.17-0.18)	0.12 (0.11-0.12)	0.23 (0.23-0.24)	0.26 (0.26-0.27)
Injury occurred nonrookie season	297	5 (2-17.15)	0.84 (0.81-0.87)	0.21 (0.19-0.22)	0.17 (0.16-0.19)	0.21(0.19-0.23)	0.25 (0.23-0.27)
Shoulder, arm, and/or elbow							
All injured NBA players	318	3 (1-9)	0.82 (0.78-0.85)	0.27 (0.25-0.29)	0.18 (0.16-0.19)	0.22 (0.20-0.24)	0.16 (0.14-0.18)
Injury occurred rookie season	39	5 (2-8)	1.13 (1.12-1.15)	0.26 (0.26-0.27)	0.12 (0.11-0.12)	0.32 (0.31-0.33)	0.44 (0.43-0.44)
Injury occurred nonrookie season	279	3 (1-8)	0.79 (0.76-0.82)	0.27 (0.25-0.28)	0.18 (0.17-0.20)	0.21 (0.19-0.23)	0.13 (0.12-0.15)
Lower leg and/or Achilles tendon							
All injured NBA players	263	4 (2-10)	0.68 (0.65-0.71)	0.15 (0.13-0.16)	0.15 (0.13-0.16)	0.25 (0.23-0.26)	0.14 (0.12-0.15)
Injury occurred rookie season	10	2.5 (1.25-11)	0.29 (0.28-0.30)	0.09 (0.08-0.09)	0.09 (0.08-0.09)	0.09 (0.08-0.09)	0.03 (0.03-0.03)
Injury occurred nonrookie season	253	4 (2-11)	0.72 (0.68-0.75)	0.16 (0.14. 0.17)	0.15 (0.14-0.17)	0.26 (0.24-0.28)	0.15 (0.13-0.16)
Head and/or neck							
All injured NBA players	113	2 (1-5)	0.29 (0.27-0.31)	0.13 (0.12. 0.15)	0.05 (0.04-0.06)	0.07 (0.06-0.09)	0.03 (0.02-0.04)
Injury occurred rookie season	12	1.5 (1-5)	0.35 (0.34-0.36)	0.17 (0.17-0.18)	0.06 (0.06-0.06)	0.12 (0.11-0.12)	0.00 (0.00-0.00)
Injury occurred nonrookie season	101	2 (1-5)	0.29 (0.26-0.31)	0.13 (0.12-0.14)	0.05 (0.04-0.06)	0.07 (0.06-0.08)	0.03 (0.03-0.04)
Concussion							
All injured NBA players	99	3 (2-6)	0.26 (0.24-0.27)	0.06 (0.05-0.07)	0.09 (0.07-0.10)	0.07 (0.06-0.08)	0.04 (0.03-0.04)
Injury occurred rookie season	19	4 (2.5-5.25)	0.55 (0.54-0.56)	0.12 (0.11-0.12)	0.15 (0.14-0.15)	0.23 (0.23-0.24)	0.06 (0.06-0.06)
Injury occurred nonrookie season	80	3 (1.75-5.25)	0.23 (0.21-0.24)	0.06 (0.05-0.07)	0.08 (0.07-0.09)	0.06 (0.05-0.07)	0.03 (0.03-0.04)

Abbreviation: AGE, athlete game exposure.

^a^ Injury severity is classified as follows: slight injury, 1 game missed; minor injury, 2 to 3 games missed; moderate injury, 4 to 13 games missed; severe injury, 14 or more games missed.

^b^ Incidence was compared between players who did or did not sustain an injury during their rookie season.

**Table 3.** Rate Ratio Based on Rookie vs Nonrookie Season by Body Region

Body part or illness	Rate ratio (95% CI)^**a**^	
	Rookie season injury	Nonrookie season injury
Ankle	1.32 (1.12 to 1.52)	0.76 (0.56 to 0.96)
Knee	0.96 (0.73 to 1.18)	1.05 (0.82 to 1.27)
Illness	1.14 (0.87 to 1.40)	0.88 (0.61 to 1.15)
Groin, hip, and/or thigh	0.78 (0.50 to 1.06)	1.28 (1.00 to 1.56)
Trunk, back, and/or buttocks	0.68 (0.34 to 1.02)	1.47 (1.12 to 1.81)
Foot and/or toe	1.29 (0.97 to 1.61)	0.78 (0.46 to 1.09)
Forearm, wrist, and/or hand	0.93 (0.53 to 1.32)	1.08 (0.68 to 1.47)
Shoulder, arm, and/or elbow	1.43 (1.10 to 1.77)	0.7 (0.36 to 1.03)
Lower leg and/or Achilles tendon	0.4 (0.00 to 1.03)	2.48 (1.85 to 3.11)
Head and/or neck	1.21 (0.61 to 1.81)	0.83 (0.23 to 1.43)
Concussion	2.39 (1.89 to 2.90)	0.42 (–0.08 to 0.92)

^a^ Rate ratio for injury that occurred during the rookie season represents the incidence rate for rookie players divided by incidence rate for veteran player stratified by body part or illness; rate ratio for injury that occurred during nonrookie season represents the incidence rate for veteran players divided by the incidence rate for rookie players stratified by body part or illness.

### Career Longevity

Professional basketball players who sustained an injury during their rookie season demonstrated an unadjusted decrease in total season played (–0.4 log years; 95% CI, –0.5 to –0.3 log years; *P* < .001), but not within the adjusted analysis (0.1 log years; 95% CI, –0.1 to 0.2 log years; *P* = .36) after adjusting for age, BMI, position, year of injury, severity of injury, and overall draft pick number. When considering draft position, for every number decrease in draft position, NBA career length decreased by −0.005 log years (95% CI, –0.006 to –0.004 log years; *P* < .001). A descriptive breakdown of career longevity and draft order by injury severity can be found in eTable 3 in the Supplement.

### Sensitivity Analysis

There was no adjusted association between sustaining a severe injury during the rookie season and career length (0.1 log year; 95% CI, –0.1 to 0.3; *P* = .14). There was no adjusted association between sustaining a lower extremity injury during rookie season and career length (0.1 log years; 95% CI, –0.1 to 0.1 log years, *P* = .46). Further sensitivity analyses and mean differences based on location of injury (eg, knee; ankle, groin, hip, and/or thigh; concussion) and severity for overall, veteran and rookie players over time can be found in eFigure 1 to eFigure 18 in the Supplement.

## Discussion

Rookie players demonstrated higher overall injury and illness incidence compared with veteran players, with the ankle associated with the most common injury location. Rookies demonstrated a higher RR of injury across multiple body regions, concussion, and illness. Career longevity demonstrated a significant unadjusted decrease among players who sustained an injury during their rookie season. However, after adjusting for confounders, no significant difference was observed. Notably, as draft position decreased, career longevity decreased.

### Injury Incidence and Risk by Location and Severity

Ankle injuries, followed by knee injuries, were the most common injury reported among all NBA players. Our findings are similar to previously reported injury incidence to the ankle and knee among NBA players.^2^ Slight differences were noted among injury incidence for the ankle and knee among rookies and veterans compared with previous literature on all NBA players.^2^ For the ankle, our findings are slightly lower among veterans but similar among rookies and slightly higher among both rookies and veterans for the knee throughout their careers. Ankle injuries, regardless of severity, can lead to ligamentous laxity, decreased proprioception, strength, and balance,^25^ which can increase the risk of recurrent ankle injuries.^25^ The impact of knee injuries varies depending on the nature and severity of the injury. Athletes with injuries that do not result in significant time loss may present with chronic issues (eg, patellar tendinopathy) or acute issues (eg, low-grade sprains) and may be managed with activity modification, short periods of rehabilitation, or rest.^26^ However, severe injuries, such as ligamentous ruptures,^27^ may require surgery and long-term rehabilitation for full recovery. Sports clinicians are encouraged to continue injury mitigation programs focusing on both the ankle and knee joints because of the high incidence and consequence of severe or subsequent ankle and knee injuries among all players. Furthermore, future research is needed to understand the slight differences among ankle and knee injury incidence between rookie and veteran players to improve injury mitigation programs.

Rookies demonstrated a higher RR of injury to multiple regions, including the ankle, foot and/or toe, shoulder, arm, and/or elbow, and head and/or neck. Further, higher RR for concussion and illness were noted among rookie athletes. The higher RR of injury across multiple regions may be related to differences in preseason conditioning status or ramp-up periods which may affect rookie athletes more than veteran players who have been in the NBA for longer.^28^ Rookies entering the NBA may be physically and psychologically underdeveloped compared with veterans.^8^ Furthermore, players entering the draft may experience a significant increase in workload because of increased training intensity and frequency of games played in an NBA season compared with a typical collegiate athlete season.^10^ Inadequate adaptation to training and workload may lead to higher injury risk and inadequate immunological responses leading to increased illness.^10^ Another possible contributor to higher RR of injury among rookies may be preprofessional exposure to sport specialization before being drafted. The association of early sport specialization on injury has been well-documented^9,29^ and may be associated with the higher RR of injury among rookie athletes across multiple regions of the body observed in our study. Our findings indicate that an emphasis on rookie development through improved injury mitigation, strength and conditioning programs, and resources for psychological and social development may be beneficial to mitigate the risk of injury.^8,9^

### Career Longevity

In the unadjusted analyses, our study demonstrated a significant decrease in the total number of seasons played among players who sustained an injury during their rookie season; however, no significant adjusted association was observed across all severity groups and career longevity. This suggests that injuries in the rookie season cannot solely account for career longevity; instead, career longevity is likely multifactorial.^30^ Furthermore, cumulative injury burden on career length vs a singular injury occurrence at a specific time point may be more influential. Injury history is a factor that has been associated with subsequent injuries across multiple locations, including anterior cruciate ligament ruptures,^31^ hamstring strains,^32^ and lateral ankle sprains.^25^ Recurrent injuries likely have a greater impact on player longevity because of cumulative pathoanatomic injury effects leading to changes in joint integrity,^25^ early-onset osteoarthritis,^33^ or increased pain,^34^ all of which may require further rehabilitation. Another notable finding was that as draft position decreased career longevity decreased. This finding may be more reflective of performance parameters than musculoskeletal injury.^35^ Although previous performance is straightforward, the quality of college attended likely reflects coaching skill and training resources available to successful draftees.^35^ Younger age may reflect the early transition to the NBA of younger, more talented players vs players who stay in college for further development.^35^ Once athletes begin playing in the NBA, players drafted lower demonstrate fewer minutes played, which impacts performance measures, and organizations often invest less in late draft picks, all of which may increase the chances of a shorter career.^22^

### Strengths and Limitations

This cohort study shares the data set, freely available research code, and custom R software package to encourage the evolution of the use of big data in sports injury surveillance and mitigation programs. This aligns with the current recommendation for open access data and code sharing^36^ to address ethical aspects of the scientific process^37^ and reproducibility of clinical research.^38^ Injury incidence and severity across multiple locations among veteran and rookie players were investigated for 12 years, increasing the generalizability of the results. Furthermore, injury data were stratified by location and severity across multiple demographic characteristics, which increased the clinical interpretability of these findings.

This study had limitations. Only NBA players were assessed, decreasing generalizability of the results to other professional leagues, amateur players, or female players. Furthermore, given the nature of the extracted data, only players who experienced an injury were included. Thus, it was not possible to compare player characteristics (eg, demographic data). The public data set did not allow for missing data to be quantified, which may impact the precision of these results; however, external data validation was performed with other publicly available data to increase result interpretability. Further, misclassification bias was possible, particularly for injuries that did not result in time loss and not recorded, which decreased the precision of the data.

Additionally, injury severity was calculated based on the first missed game. Injuries sustained in a previous game, practice, or training session could not be accurately determined, decreasing the precision of the severity and temporal analyses. Days missed to classify injury severity has been previously used^2,39^; however, because publicly available data were used, it was determined that using games missed would decrease bias. Furthermore, injury data were limited and inconsistent in the preseason time frame and were not included in this study, which may impact a proportion of injuries that were not accounted for, particularly among rookie athletes. This timeframe often reflects a higher incidence of injury because of issues with conditioning or workload management and warrants further investigation, especially among rookie athletes.^28^ Improved methods for capturing precise injury dates were needed among publicly available data sites to allow for comparison of practice vs game injury rates, a comparison that was not within the scope of this study. Some injuries were reported to the nearest anatomical body part, with specific injury classification not possible (eg, knee injury vs right knee meniscal tear), which decreased the clinical interpretability of these findings. Poisson regression models could not account for the cumulative injury burden on career length vs a singular injury occurrence at a specific time point.^22^ As cumulative injury burden can potentially diminish career longevity^25,34^; this decreased the precision of these results.

## Conclusions

In this cohort study of NBA athletes, rookie basketball players demonstrated a higher overall injury and illness incidence, with the ankle representing the most common injury. Rookie athletes demonstrated a higher RR of injury across multiple regions, which may reflect maturational differences compared with the rest of the NBA. Players who sustained an injury during their rookie season demonstrated an unadjusted decrease in the total number of seasons played but not within the adjusted analysis. This finding may reflect the association of cumulative injury burden vs injury at a specific time point.

## Notes

Supplement. **eAppendix 1.** Details on Data Scraping Methodology **eAppendix 2.** Data Extraction R Code **eTable 1.** Temporal Trends of Injury Incidence Severity **eTable 2.** Temporal Trends Overall **eTable 3.** Temporal Trends by Severity **eTable 4.** Player Characteristics by Injury Severity and Injury Status Rookie Year **eFigure 1.** Sensitivity Analyses **eFigure 2.** 4-Year Time Loss Injury **eFigure 3.** 4-Year Ankle Time Loss Injury **eFigure 4.** 4-Year Knee Time Loss Injury **eFigure 5.** 4-Year Groin/Hip/Thigh Time Loss Injury **eFigure 6**. 4-Year Concussion Time Loss Injury **eFigure 7.** 4-Year Time Loss Injury of Veteran Players **eFigure 8.** 4-Year Time Loss Injury of Rookie Players **eFigure 9.** 4 Year Time Loss for Severity of Veteran Players **eFigure 10.** 4-Year Time Loss for Severity of Rookie Players **eFigure 11.** 4-Year Ankle Time Loss of Veteran Players **eFigure 12.** 4-Year Ankle Time Loss of Rookie Players **eFigure 13.** 4-Year Knee Time Loss of Veteran Players **eFigure 14.** 4-Year Knee Time Loss of Rookie Players **eFigure 15.** 4-Year Groin/Hip/Thigh Time Loss of Veteran Players **eFigure 16.** 4-Year Groin/Hip/Thigh Time Loss of Rookie Players **eFigure 17.** 4-Year Concussion Time Loss of Veteran Players **eFigure 18.** 4-Year Concussion Time Loss of Rookie Players **eTable 5.** Mean Difference of Injury Status by Location, Severity, and Rookie Year **eTable 6.** Initial vs Subsequent Injury Incidence by Body Part **eTable 7.** Poisson Regression Results **eTable 8.** Rate Ratios for Initial vs Subsequent Injuries in Rookie vs Veteran Athletes
