# Supplementary material for: Characterization of Rookie Season Injury and Illness and Career Longevity Among National Basketball Association Players
Source: JAMA Netw Open. 2021 Oct 4;4(10):e2128199. doi: 10.1001/jamanetworkopen.2021.28199 (PMC8491104; doi:10.1001/jamanetworkopen.2021.28199)
Supplement: Supplement. — eAppendix 1. Details on Data Scraping Methodology eAppendix 2. Data Extraction R Code eTable 1. Temporal Trends of Injury Incidence Severity eTable 2. Temporal Trends Overall eTable 3. Temporal Trends by Severity eTable 4. Player Characteristics by Injury Severity and Injury Status Rookie Year eFigure 1. Sensitivity Analyses eFigure 2. 4-Year Time Loss Injury eFigure 3. 4-Year Ankle Time Loss Injury eFigure 4. 4-Year Knee Time Loss Injury eFigure 5. 4-Year Groin/Hip/Thigh Time Loss Injury eFigure 6. 4-Year Concussion Time Loss Injury eFigure 7. 4-Year Time Loss Injury of Veteran Players eFigure 8. 4-Year Time Loss Injury of Rookie Players eFigure 9. 4 Year Time Loss for Severity of Veteran Players eFigure 10. 4-Year Time Loss for Severity of Rookie Players eFigure 11. 4-Year Ankle Time Loss of Veteran Players eFigure 12. 4-Year Ankle Time Loss of Rookie Players eFigure 13. 4-Year Knee Time Loss of Veteran Players eFigure 14. 4-Year Knee Time Loss of Rookie Players eFigure 15. 4-Year Groin/Hip/Thigh Time Loss of Veteran Players eFigure 16. 4-Year Groin/Hip/Thigh Time Loss of Rookie Players eFigure 17. 4-Year Concussion Time Loss of Veteran Players eFigure 18. 4-Year Concussion Time Loss of Rookie Players eTable 5. Mean Difference of Injury Status by Location, Severity, and Rookie Year eTable 6. Initial vs Subsequent Injury Incidence by Body Part eTable 7. Poisson Regression Results eTable 8. Rate Ratios for Initial vs Subsequent Injuries in Rookie vs Veteran Athletes [file jamanetwopen-e2128199-s001.pdf]

Supplemental Online Content

Martin CL, Arundale AJH, Kluzek S, et al. Characterization of rookie season injury and illness and carerer longevity among National Basketball Association players. *JAMA Netw Open.* 2021;4(10):e2128199. doi:10.1001/jamanetworkopen.2021.28199

eAppendix 1. Details on Data Scraping Methodology
eAppendix 2. Data Extraction R Code
eTable 1. Temporal Trends of Injury Incidence Severity
eTable 2. Temporal Trends Overall
eTable 3. Temporal Trends by Severity
eTable 4. Player Characteristics by Injury Severity and Injury Status Rookie Year
eFigure 1. Sensitivity Analyses
eFigure 2. 4-Year Time Loss Injury
eFigure 3. 4-Year Ankle Time Loss Injury
eFigure 4. 4-Year Knee Time Loss Injury
eFigure 5. 4-Year Groin/Hip/Thigh Time Loss Injury
eFigure 6. 4-Year Concussion Time Loss Injury
eFigure 7. 4-Year Time Loss Injury of Veteran Players
eFigure 8. 4-Year Time Loss Injury of Rookie Players
eFigure 9. 4-Year Time Loss for Severity of Veteran Players
eFigure 10. 4-Year Time Loss for Severity of Rookie Players
eFigure 11. 4-Year Ankle Time Loss of Veteran Players
eFigure 12. 4-Year Ankle Time Loss of Rookie Players
eFigure 13. 4-Year Knee Time Loss of Veteran Players
eFigure 14. 4-Year Knee Time Loss of Rookie Players
eFigure 15. 4-Year Groin/Hip/Thigh Time Loss of Veteran Players
eFigure 16. 4-Year Groin/Hip/Thigh Time Loss of Rookie Players
eFigure 17. 4-Year Concussion Time Loss of Veteran Players
eFigure 18. 4-Year Concussion Time Loss of Rookie Players
eTable 5. Mean Difference of Injury Status by Location, Severity, and Rookie Year
eTable 6. Initial vs Subsequent Injury Incidence by Body Part
eTable 7. Poisson Regression Results
eTable 8. Rate Ratios for Initial vs Subsequent Injuries in Rookie vs Veteran Athletes

This supplemental material has been provided by the authors to give readers additional information about their work.

© 2021 Martin CL et al. *JAMA Network Open.*

eAppendix 1. Details on Data Scraping Methodology

Data were extracted through a reproducible programmed process, known as 'data scraping,' in which a programming language retrieves data from online publicly available depositories (for example websites), into a readable output (Landers RN et al 2016). Three large online resourceswere utilized to create a combined dataset for this study: https://www.prosportstransactions.com, https://www.basketball-reference.com, and https://stats.nba.com. The website 'Pro Sport Transactions' documents every professional North American sport transaction, including trades, the draft, free agent signings, injuries, and disciplinary and legal actions. The website Basketball-Reference.com provides current and historical basketball statistics for the NBA, ABA (American Basketball Association, predecessor to the NBA), WNBA (Women's NBA), and the Euroleague. The NBA website (stats.nba.com) is the official website for NBA statistics.

Landers RN, Brusso RC, Cavanaugh KJ, Collmus AB. A primer on theory-driven web scraping:Automatic extraction of big data from the Internet for use in psychological research. *Psych Method.* 2016;21(4):475.

© 2021 Martin CL et al. *JAMA Network Open.*

eAppendix 2. Data Extraction R Code
The freely available online code and data repository: GitHub R Package"tylerferguson/NBAinjuries."

```
#Data
Scraping
library(r
obotstxt
)
librar
y(xml
2)
librar
y(rve
st)
librar
y(stri
ngr)
librar
y(dpl
yr)
librar
y(tidy
r)
librar
y(pur
rr)

#Table iterations
#/html/body/div[4]/table[1]
#/html/body/div[4]/table[1]
#/html/body/div[4]/table[1]
#Same Table HTML code for every table

#Web page
#https://www.prosportstransactions.com/baseball/Search/SearchResults.php?Player=&Team=&BeginD
ate=&EndDate=&InjuriesChkBx=yes&submit=Search&start=525
#https://www.prosportstransactions.com/baseball/Search/SearchResults.php?Player=&Team=&BeginD
ate=&EndDate=&InjuriesChkBx=yes&submit=Search&start=550
#https://www.prosportstransactions.com/baseball/Search/SearchResults.php?Player=&Team=&BeginD
ate=&EndDate=&InjuriesChkBx=yes&submit=Search&start=575
#https://www.prosportstransactions.com/baseball/Search/SearchResults.php?Player=&Team=&BeginD
ate=&EndDate=&InjuriesChkBx=yes&submit=Search&start=600
#Web pages go up by 25
```

© 2021 Martin CL et al. *JAMA Network Open.*

```
#Multiple pages:
url_base <-
 "https://www.prosportstransactions.com/baseball/Search/SearchResults.php?Player=&Team=&Begin
 D ate=&EndDate=&InjuriesChkBx=yes&submit=Search&start=%d"

map_df(seq(0,100, 25),
  function(i) {
  print(sprintf(url_base, i))
  data.frame(html_nodes(read_html(sprintf(url_base, i)), xpath =
    '/html/body/div[4]/table[1]') %>%html_table(header = T))

}) -

>

te

sti

ng

#

w

or

ks

testing <-
  testing %>%
  mutate(
    Acquired = substring(Acquired,
    3), Relinquished =
    substring(Relinquished, 3)
  #Basketball)
url_basket <-
 "https://www.prosportstransactions.com/basketball/Search/SearchResults.php?Player=&Team=&Begi
 nDate=&EndDate=&InjuriesChkBx=yes&Submit=Search&start=%d"

map_df(seq(0,27900, 25),
  function(i) {print(i)
  data.frame(html_nodes(read_html(sprintf(url_basket, i)), xpath =
          '/html/body/div[4]/table[1]') %>%html_table(header = T))

}) -> basketball_injury
```

© 2021 Martin CL et al. *JAMA Network Open.*

```
basketball_injury <-
  basketball_injury %>%mutate(
    Acquired = substring(Acquired,
    3), Relinquished =
    substring(Relinquished, 3)
  )

  write.csv(basketball_injury, "/Users/student/Desktop/basketball_injury.csv")
```

© 2021 Martin CL et al. *JAMA Network Open.*

eTable 1. Temporal Trends of Injury Incidence Severity

Year	Injury Incidence	Slight Injury Severity Incidence	Minor Injury Severity Incidence	Moderate Injury Severity Incidence	Severe Injury Severity Incidence
All NBA Players					
2007-2008	14.98	4.31	3.66	3.86	1.61
2008-2009	15.89	3.78	3.81	3.89	2.32
2009-2010	16.53	4.20	3.30	4.00	2.53
Year	Injury Incidence	Slight Injury Severity Incidence	Minor Injury Severity Incidence	Moderate Injury Severity Incidence	Severe Injury Severity Incidence
2011-2012	22.75	5.58	3.88	4.53	4.20
2012-2013	15.21	5.00	3.71	3.21	1.62
2013-2014	14.79	4.18	3.55	3.73	1.91
2014-2015	14.85	4.61	3.86	3.13	1.79
2015-2016	15.00	4.95	3.27	3.33	1.90
2016-2017	14.71	4.15	3.78	3.60	1.80
2017-2018	15.63	4.84	2.97	3.37	2.52
2018-2019	14.54	4.53	3.38	3.54	1.81
Veteran Players					
2007-2008	13.22	4.38	3.62	3.75	1.47
2008-2009	13.64	3.83	3.99	3.75	2.08
2009-2010	14.01	4.29	3.27	3.99	2.46
2010-2011	13.67	3.53	4.04	3.96	2.14
2011-2012	17.84	5.54	3.88	4.51	3.92
2012-2013	13.49	5.08	3.68	3.12	1.60
2013-2014	13.38	4.21	3.44	3.76	1.97
2014-2015	13.44	4.61	3.90	3.14	1.79
2015-2016	13.41	4.94	3.29	3.37	1.81
2016-2017	13.35	4.16	3.82	3.59	1.77
2017-2018	13.63	4.94	3.06	3.22	2.42
2018-2019	13.24	4.59	3.25	3.62	1.78
All NBA Players					
2007-2008	14.98	4.31	3.66	3.86	1.61
2008-2009	15.89	3.78	3.81	3.89	2.32
2009-2010	16.53	4.20	3.30	4.00	2.53
2010-2011	15.45	3.69	3.87	3.95	2.12
2011-2012	16.68	4.32	3.01	3.51	3.26

© 2021 Martin CL et al. *JAMA Network Open.*

2012-2013	20.10	6.38	4.74	4.10	2.07
2013-2014	14.79	4.18	3.55	3.73	1.91
2014-2015	14.85	4.61	3.86	3.13	1.79
2015-2016	15.00	4.95	3.27	3.33	1.90
2016-2017	14.71	4.15	3.78	3.60	1.80
2017-2018	15.63	4.84	2.97	3.37	2.52
2018-2019	14.54	4.53	3.38	3.54	1.81
Injury incidence is reported per 1000 athlete game exposures					
Slight Injury = 1 game missed; Minor Injury = 2-3 games missed; Moderate Injury = 4-13 games missed; Severe Injury = 14+ games missed					

© 2021 Martin CL et al. *JAMA Network Open.*

eTable 2. Temporal Trends Overall

**Overall Injury/Illness Incidence**			
	All NBA Players	Rookies	Veterans
2007-2008	14.98	18.33	13.22
2008-2009	15.89	15.59	13.64
2009-2010	16.53	14.29	14.01
2010-2011	15.45	12.95	13.67
2011-2012	22.75	24.64	17.84
2012-2013	15.21	14.28	13.49
2013-2014	14.79	13.25	13.38
2014-2015	14.85	13.09	13.44
2015-2016	15.00	13.88	13.41
2016-2017	14.71	13.22	13.35
2017-2018	15.63	14.27	13.63
2018-2019	14.54	13.45	13.24
**Temporal Ankle Injury Incidence**			
	All NBA Players	Rookies	Veterans
2007-2008	2.74	5.50	2.61
2008-2009	2.24	3.21	2.16
2009-2010	2.33	3.69	2.21
2010-2011	2.99	8.74	2.68
2011-2012	2.74	1.84	2.79
2012-2013	3.49	2.39	3.56
2013-2014	2.75	3.44	2.68
2014-2015	2.38	3.55	2.21
2015-2016	1.95	2.55	1.90
2016-2017	2.19	2.12	2.20
2017-2018	2.80	2.80	2.80
2018-2019	2.36	1.82	2.44
**Temporal Concussion Injury Incidence**			
	All NBA Players	Rookies	Veterans
2007-2008	0.20	0.92	0.17
2008-2009	0.22	0.00	0.24
2009-2010	0.08	0.00	0.08
2010-2011	0.15	0.00	0.16
**Temporal Concussion Injury Incidence (continued)**			
	All NBA Players	Rookies	Veterans

© 2021 Martin CL et al. *JAMA Network Open.*

2012-2013	0.20	0.00	0.21
2013-2014	0.05	0.26	0.03
2014-2015	0.31	0.67	0.26
2015-2016	0.36	0.85	0.31
2016-2017	0.31	0.79	0.26
2017-2018	0.37	0.76	0.32
2018-2019	0.27	0.91	0.19
**Temporal Foot/Toe Injury Incidence**			
	All NBA Players	Rookies	Veterans
2007-2008	0.81	1.83	0.76
2008-2009	1.05	1.83	0.98
2009-2010	0.82	0.92	0.81
2010-2011	1.27	1.85	1.23
2011-2012	1.37	1.59	1.36
2012-2013	0.95	0.00	1.01
2013-2014	0.95	1.85	0.85
2014-2015	0.64	0.67	0.64
2015-2016	0.96	1.42	0.92
2016-2017	1.00	0.53	1.06
2017-2018	0.96	0.76	0.99
2018-2019	1.21	2.28	1.06
**Temporal Forearm/hand/wrist Injury Incidence**			
	All NBA Players	Rookies	Veterans
2007-2008	1.09	0.00	1.14
2008-2009	1.12	0.46	1.18
2009-2010	1.09	1.38	1.06
2010-2011	0.67	0.00	0.71
2011-2012	0.93	0.79	0.94
2012-2013	0.59	0.92	0.56
2013-2014	0.71	1.59	0.62
2014-2015	0.87	0.89	0.86
2015-2016	0.69	0.00	0.75
2016-2017	0.98	1.06	0.97
2017-2018	0.88	1.02	0.86
2018-2019	0.63	0.46	0.66
**Temporal Groin/Hip/Thigh Injury Incidence**			

© 2021 Martin CL et al. *JAMA Network Open.*

	All NBA Players	Rookies	Veterans
2007-2008	2.09	1.83	2.11
2008-2009	1.98	1.38	2.04
2009-2010	1.40	1.38	1.40
2010-2011	1.86	1.85	1.86
2011-2012	2.87	1.59	2.94
2012-2013	1.79	2.76	1.72
2013-2014	1.38	0.53	1.47
2014-2015	2.04	1.55	2.11
2015-2016	1.98	0.57	2.12
2016-2017	2.29	1.85	2.34
2017-2018	1.95	2.29	1.91
2018-2019	2.14	1.82	2.19
**Temporal Head/Neck Injury Incidence**			
	All NBA Players	Rookies	Veterans
2007-2008	0.24	0.92	0.21
2008-2009	0.34	0.00	0.37
2009-2010	0.51	1.38	0.42
2010-2011	0.24	0.00	0.32
2011-2012	0.22	0.00	0.26
2012-2013	0.29	0.26	0.24
2013-2014	0.16	0.67	0.15
2014-2015	0.25	0.00	0.19
2015-2016	0.38	0.00	0.42
2016-2017	0.23	0.51	0.26
2017-2018	0.34	0.23	0.32
2018-2019	0.30	0.23	0.31
**Temporal Illness Injury Incidence**			
	All NBA Players	Rookies	Veterans
2007-2008	1.41	1.83	1.39
2008-2009	1.61	3.21	1.47
2009-2010	1.91	0.92	2.00
2010-2011	1.60	1.23	1.63
2011-2012	1.98	4.77	1.83
2012-2013	1.76	2.30	1.72
**Temporal Illness Injury Incidence (Continued)**			
	All NBA Players	Rookies	Veterans

© 2021 Martin CL et al. *JAMA Network Open.*

2013-2014	1.64	1.32	1.68
2014-2015	1.65	2.00	1.60
2015-2016	1.45	1.42	1.45
2016-2017	1.54	2.64	1.43
2017-2018	0.93	0.51	0.99
2018-2019	1.29	1.14	1.31
**Temporal Knee Injury Incidence**			
	All NBA Players	Rookies	Veterans
2007-2008	2.09	3.67	2.02
2008-2009	2.21	1.83	2.24
2009-2010	2.33	2.31	2.34
2010-2011	2.72	0.62	2.85
2011-2012	3.11	2.38	3.15
2012-2013	2.37	3.22	2.32
2013-2014	2.86	2.38	2.91
2014-2015	2.01	0.67	2.21
2015-2016	2.49	3.40	2.40
2016-2017	2.52	2.38	2.54
2017-2018	2.69	3.31	2.61
2018-2019	2.66	2.96	2.62
**Temporal Lower leg/Achilles tendon Incidence**			
	All NBA Players	Rookies	Veterans
2007-2008	0.56	0.00	0.59
2008-2009	0.45	0.46	0.45
2009-2010	0.70	0.00	0.76
2010-2011	0.56	0.62	0.55
2011-2012	0.97	0.00	1.02
2012-2013	0.64	0.46	0.65
2013-2014	0.77	0.26	0.82
2014-2015	0.73	0.00	0.83
2015-2016	0.71	0.28	0.75
2016-2017	0.82	0.79	0.83
2017-2018	0.65	0.25	0.70
2018-2019	0.52	0.23	0.56
**Temporal Shoulder/Arm/Elbow Incidence**			
	All NBA Players	Rookies	Veterans

© 2021 Martin CL et al. *JAMA Network Open.*

2007-2008	0.68	0.92	0.67
2008-2009	0.79	1.38	0.73
2009-2010	0.66	0.92	0.64
2010-2011	0.63	0.62	0.63
2011-2012	1.25	2.38	1.19
2012-2013	0.98	2.30	0.89
2013-2014	0.64	0.26	0.68
2014-2015	0.98	1.11	0.96
2015-2016	1.04	1.98	0.95
2016-2017	0.67	0.53	0.68
2017-2018	0.96	1.27	0.92
2018-2019	0.55	0.91	0.50
**Temporal Trunk/Back/Buttock**			
	All Injured NBA Players	Rookies	Veterans
2007-2008	1.53	0.92	1.56
2008-2009	1.80	1.83	1.79
2009-2010	2.22	1.38	2.29
2010-2011	1.56	0.62	1.63
2011-2012	1.94	1.59	1.96
2012-2013	1.31	0.46	1.37
2013-2014	1.46	1.06	1.50
2014-2015	1.54	1.33	1.57
2015-2016	1.45	1.42	1.45
2016-2017	0.77	0.53	0.80
2017-2018	1.16	0.76	1.21
2018-2019	1.32	0.68	1.41

Injury incidence is reported per 1000 athlete game exposures

© 2021 Martin CL et al. *JAMA Network Open.*

eTable 3. Temporal Trends by Severity

**Temporal Trends for Severe Injury/Illness Incidence**			
	All NBA Players	Rookies	Veterans
2007-2008	1.61	4.58	1.47
2008-2009	2.32	5.04	2.08
2009-2010	2.53	3.23	2.46
2010-2011	2.12	1.85	2.14
**Temporal Trends for Severe Injury/Illness Incidence (Continued)**			
	All NBA Players	Rookies	Veterans
2011-2012	4.20	9.54	3.92
2012-2013	1.62	1.84	1.60
2013-2014	1.91	1.32	1.97
2014-2015	1.79	1.77	1.79
2015-2016	1.90	2.83	1.81
2016-2017	1.80	2.12	1.77
2017-2018	2.52	3.31	2.42
2018-2019	1.81	2.05	1.78
**Temporal Trends for Moderate Injury/Illness Incidence**			
	All NBA Players	Rookies	Veterans
2007-2008	3.86	6.42	3.75
2008-2009	3.89	5.50	3.75
2009-2010	4.00	4.15	3.99
2010-2011	3.95	3.70	3.96
2011-2012	4.53	4.77	4.51
2012-2013	3.21	4.61	3.12
2013-2014	3.73	3.44	3.76
2014-2015	3.13	3.11	3.14
2015-2016	3.33	2.83	3.37
2016-2017	3.60	3.70	3.59
2017-2018	3.37	4.59	3.22
2018-2019	3.54	2.96	3.62
**Temporal Trends for Minor Injury/Illness Incidence**			
	All NBA Players	Rookies	Veterans
2007-2008	3.66	4.58	3.62
2008-2009	3.81	1.83	3.99
2009-2010	3.30	3.69	3.27

© 2021 Martin CL et al. *JAMA Network Open.*

2010-2011	3.87	1.23	4.04
2011-2012	3.88	3.97	3.88
2012-2013	3.71	4.15	3.68
2013-2014	3.55	4.50	3.44
2014-2015	3.86	3.55	3.90
2015-2016	3.27	3.12	3.29
2016-2017	3.78	3.44	3.82
2017-2018	2.97	2.29	3.06
**Temporal Trends for Minor Injury/Illness Incidence**			
	All NBA Players	Rookies	Veterans
2018-2019	3.38	4.33	3.25
**Combined Temporal Trends for Slight Injury/Illness Incidence**			
	All NBA Players	Rookies	Veterans
2007-2008	4.31	2.75	4.38
2008-2009	3.78	3.21	3.83
2009-2010	4.20	3.23	4.29
2010-2011	3.69	6.17	3.53
2011-2012	5.58	6.36	5.54
2012-2013	5.00	3.68	5.08
2013-2014	4.18	3.97	4.21
2014-2015	4.61	4.66	4.61
2015-2016	4.95	5.10	4.94
2016-2017	4.15	3.97	4.16
2017-2018	4.84	4.08	4.94
2018-2019	4.53	4.10	4.59

Injury incidence is reported per 1000 athlete game exposures
Slight Injury = 1 game missed; Minor Injury = 2-3 games missed; Moderate Injury = 4-13 games missed; Severe Injury = 14+ games missed

© 2021 Martin CL et al. *JAMA Network Open.*

eTable 4. Player Characteristics by Injury Severity and Injury Status Rookie Year

Injury Severity	Age	BMI (kg/m2)	Draft Order	Seasons Played
**Slight**				
All slight severity injuries	27.2 (4.1)	25.1 (1.6)	15 (5, 27)	7.8(4.3)
Injury occurred rookie season	22.6 (2.2)	24.9 (1.9)	15 (5, 29)	5.8 (2.4)
Injury occurred as a veteran	27.6 (4.0)	25.1 (1.6)	15 (5, 26.25)	8.3 (4.4)
**Minor**				
All minor severity injuries	27.0 (4.1)	25.0 (1.6)	16 (6, 27)	7.4 (4.3)
Injury occurred rookie season	22.7 (2.5)	24.7 (1.6)	22 (10, 33)	4.6 (2.4)
Injury occurred as a veteran	27.4 (3.9)	25.0 (1.6)	15 (6, 27)	8.1 (4.8)
**Moderate**				
All moderate severity injuries	27.1 (4.2)	24.9 (1.8)	17 (7, 30)	7.2 (4.6)
Injury occurred rookie season	22.8 (1.8)	25.0 (2.0)	24 (8.5, 39)	5.0 (2.8
Injury occurred as a veteran	27.5 (4.2)	24.9 (1.7)	16 (7, 28)	7.8 (5.1)
**Severe**				
All severe severity injuries	26.8 (4.4)	25.0 (1.8)	18 (7.25, 30)	5.6 (4.4)
Injury occurred rookie season	22.8 (2.2)	24.6 (1.8)	22.5 (13.5, 34)	4.5 (3.1)
Injury occurred as a veteran	27.3 (4.3)	25.0 (1.8)	17 (7, 29.75)	6.2 (4.7)

Results are reported as mean (standard deviation) or median (interquartile range)

© 2021 Martin CL et al. *JAMA Network Open.*

eFigure 1. Sensitivity Analyses

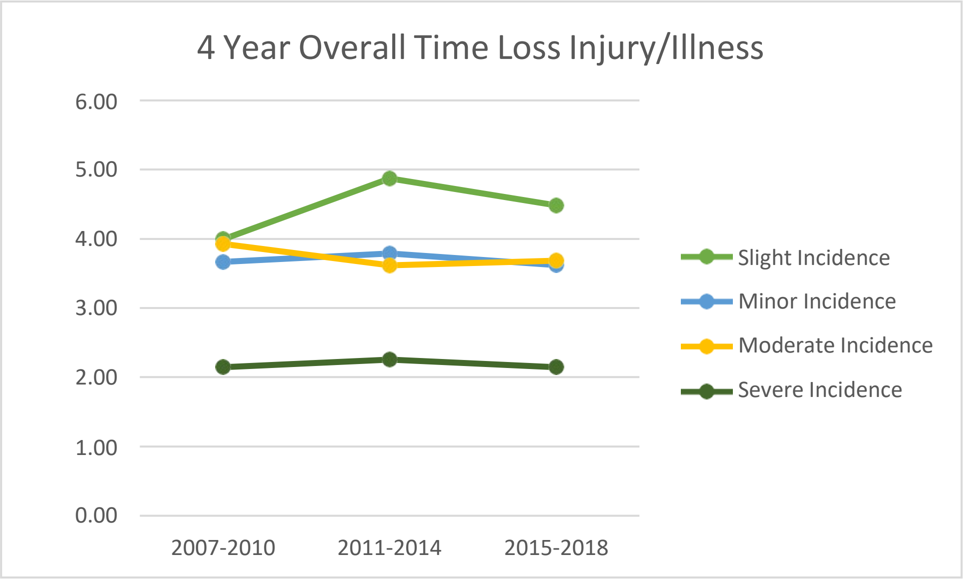

© 2021 Martin CL et al. *JAMA Network Open.*

eFigure 2. 4-Year Time Loss Injury

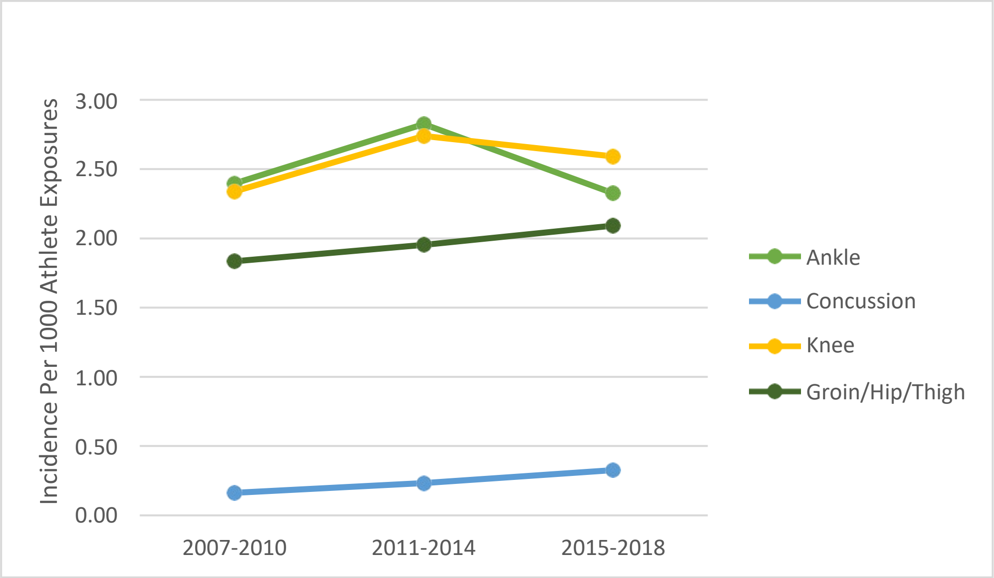

© 2021 Martin CL et al. *JAMA Network Open.*

eFigure 3. 4-Year Ankle Time Loss Injury

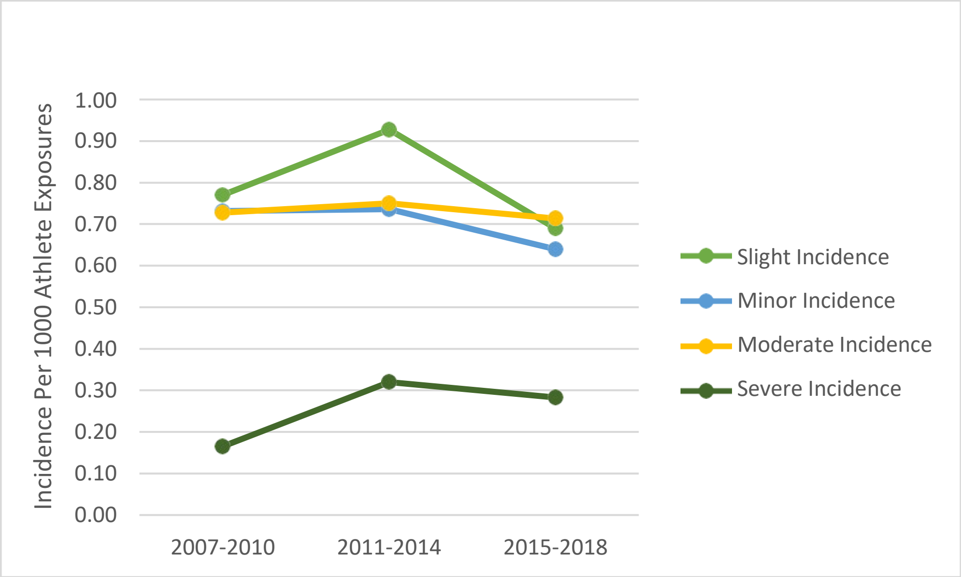

© 2021 Martin CL et al. *JAMA Network Open.*

eFigure 4. 4-Year Knee Time Loss Injury

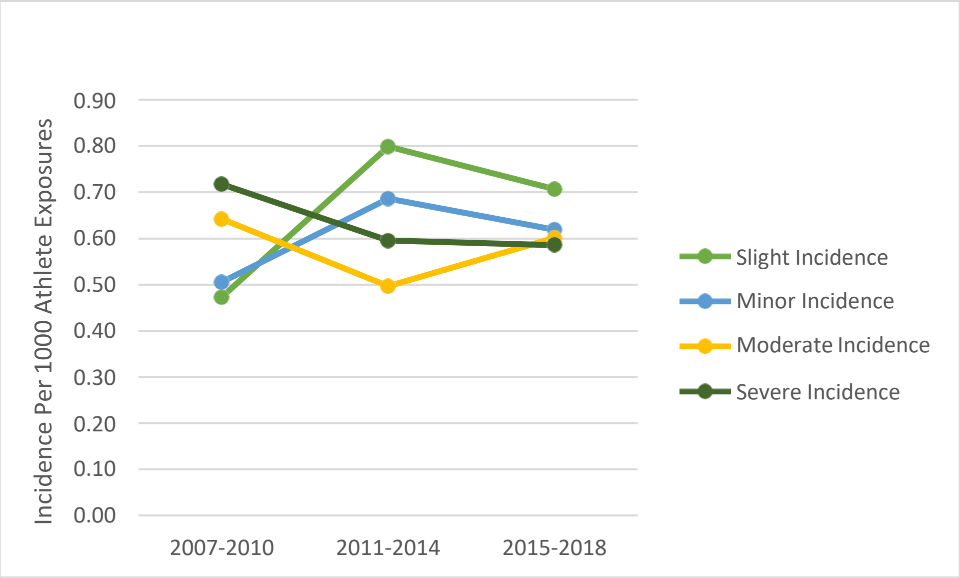

© 2021 Martin CL et al. *JAMA Network Open.*

eFigure 5. 4 Year Groin/Hip/Thigh Time Loss Injury

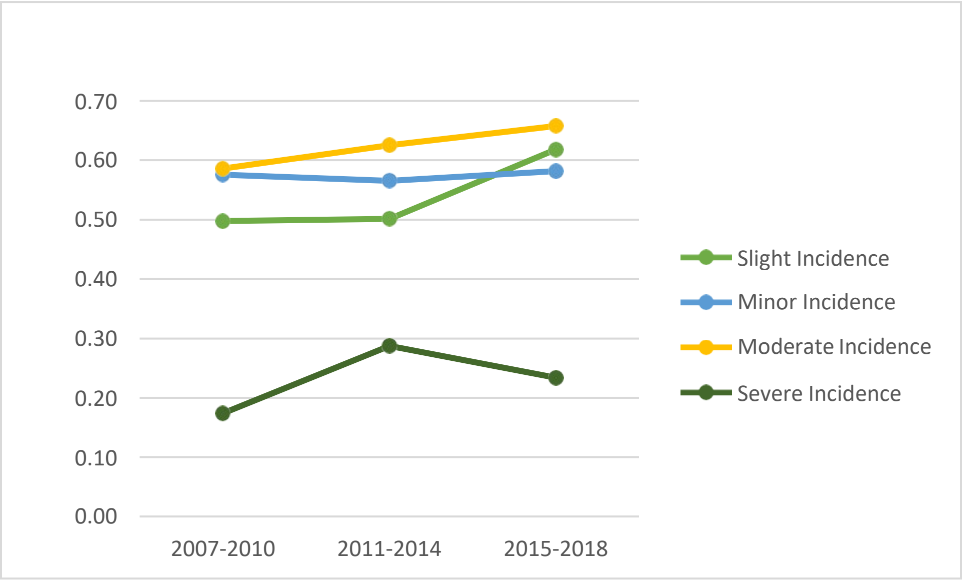

© 2021 Martin CL et al. *JAMA Network Open.*

eFigure 6. 4-Year Concussion Time Loss Injury

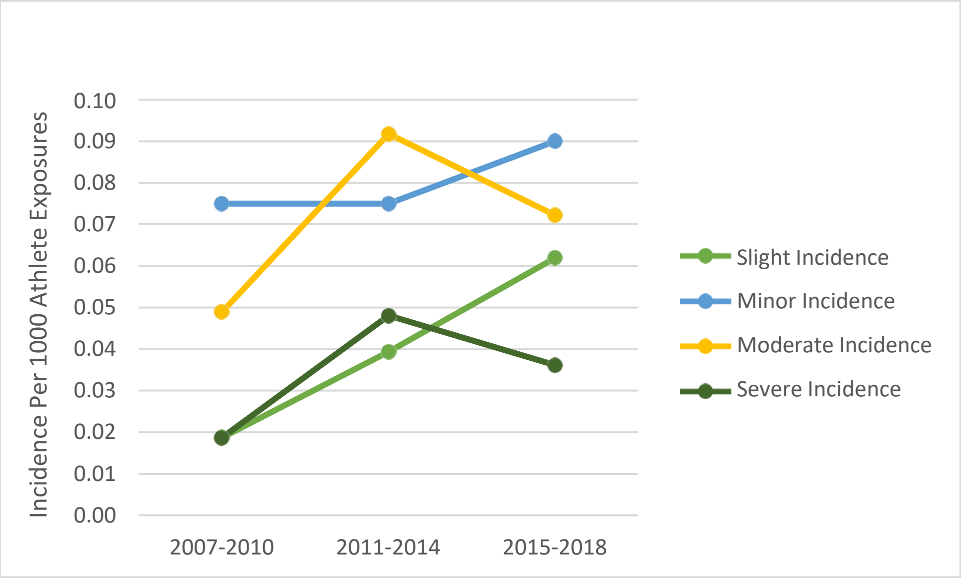

© 2021 Martin CL et al. *JAMA Network Open.*

eFigure 7. 4-Year Time Loss Injury of Veteran Players

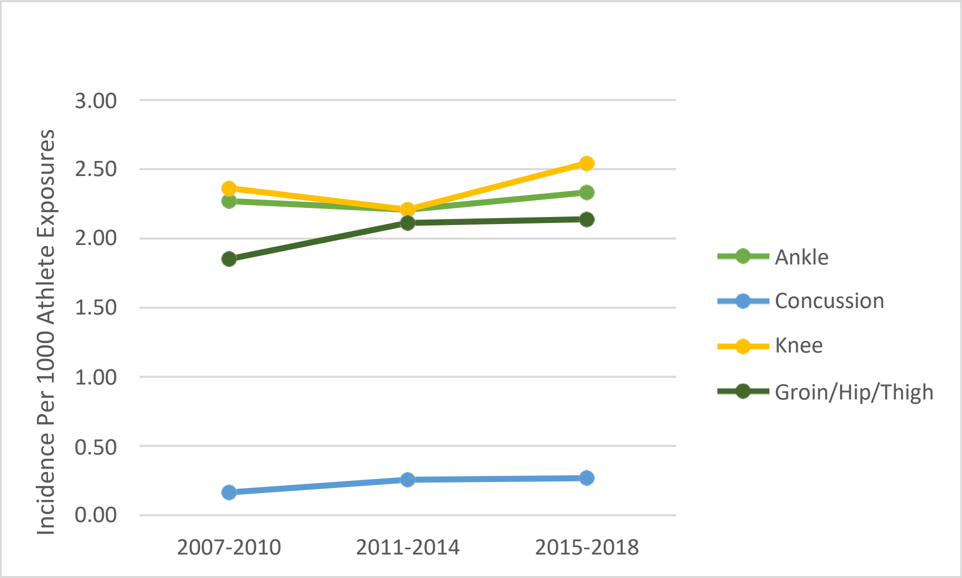

© 2021 Martin CL et al. *JAMA Network Open.*

eFigure 8. 4-Year Time Loss Injury of Rookie Players

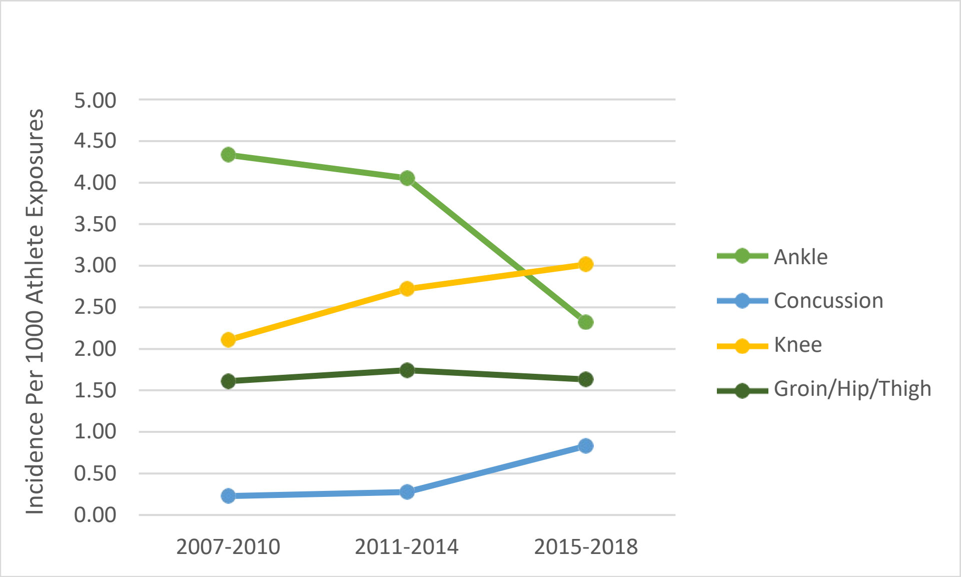

eFigure 9. 4 Year Time Loss for Severity of Veteran Players

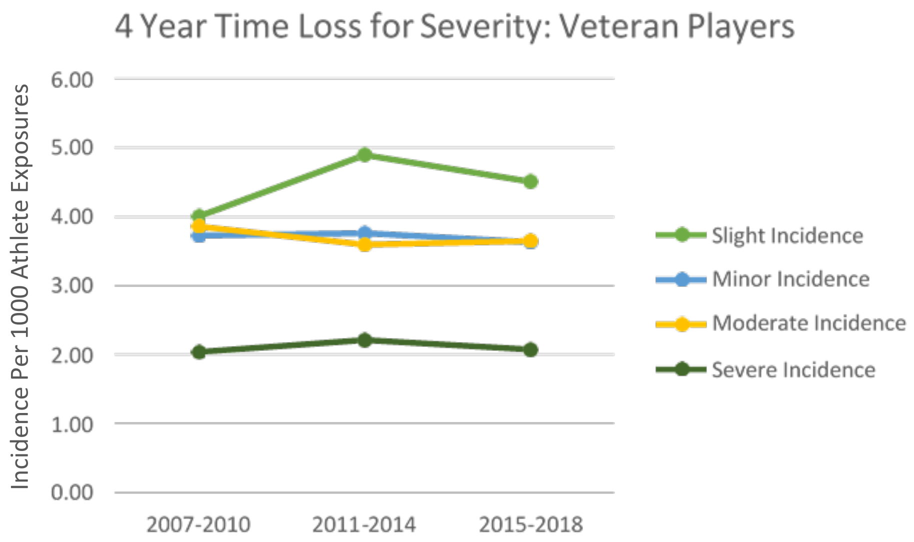

© 2021 Martin CL et al. *JAMA Network Open.*

eFigure 10. 4-Year Time Loss for Severity of Rookie Players.

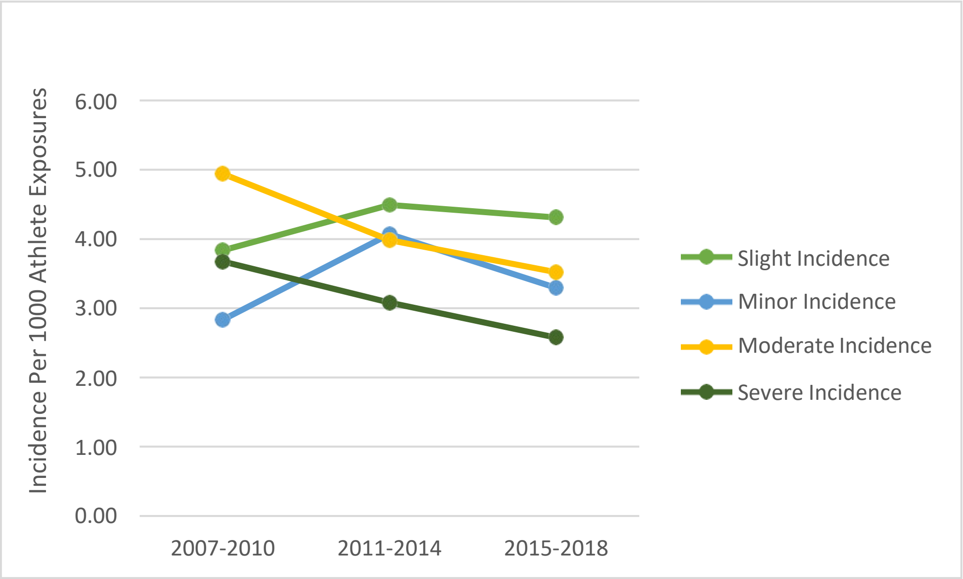

© 2021 Martin CL et al. *JAMA Network Open.*

eFigure 11. 4-Year Ankle Time Loss of Veteran Players

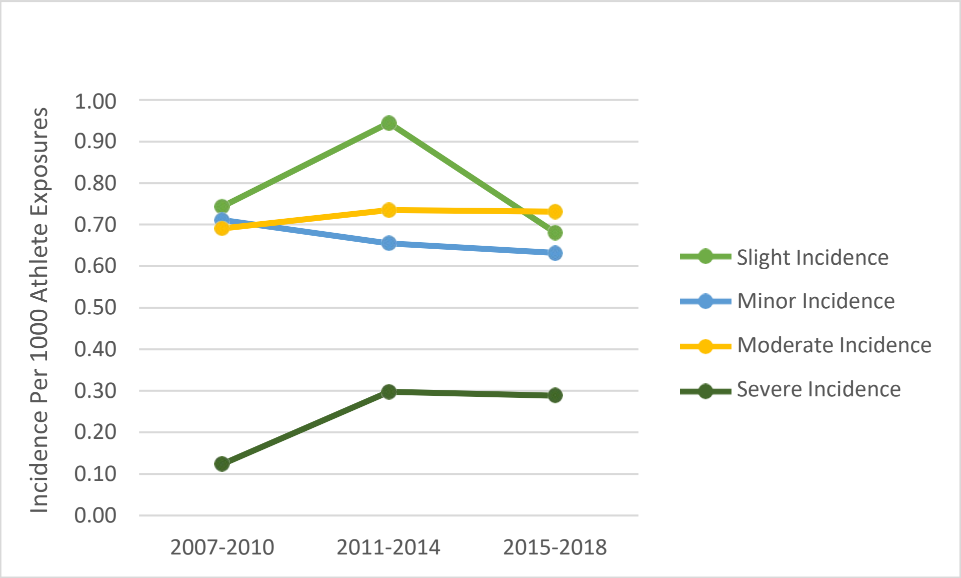

© 2021 Martin CL et al. *JAMA Network Open.*

eFigure 12. 4-Year Ankle Time Loss of Rookie Players

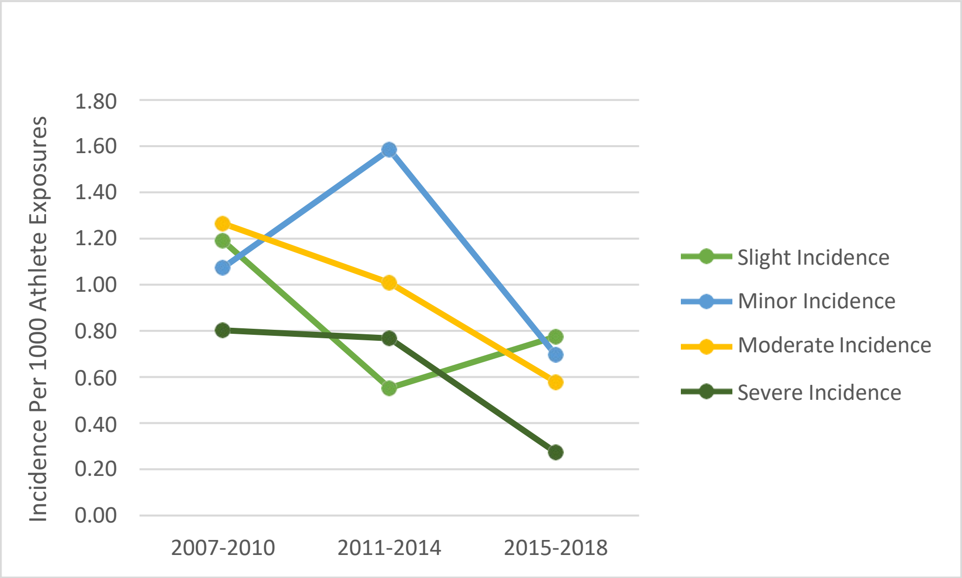

© 2021 Martin CL et al. *JAMA Network Open.*

eFigure 13. 4-Year Knee Time Loss of Veteran Players

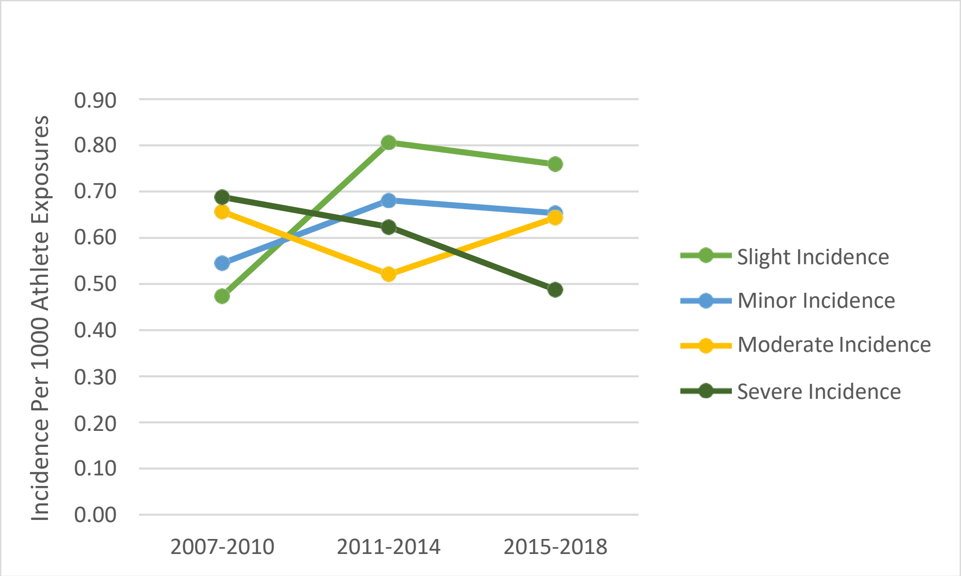

© 2021 Martin CL et al. *JAMA Network Open.*

eFigure 14. 4-Year Knee Time Loss of Rookie Players

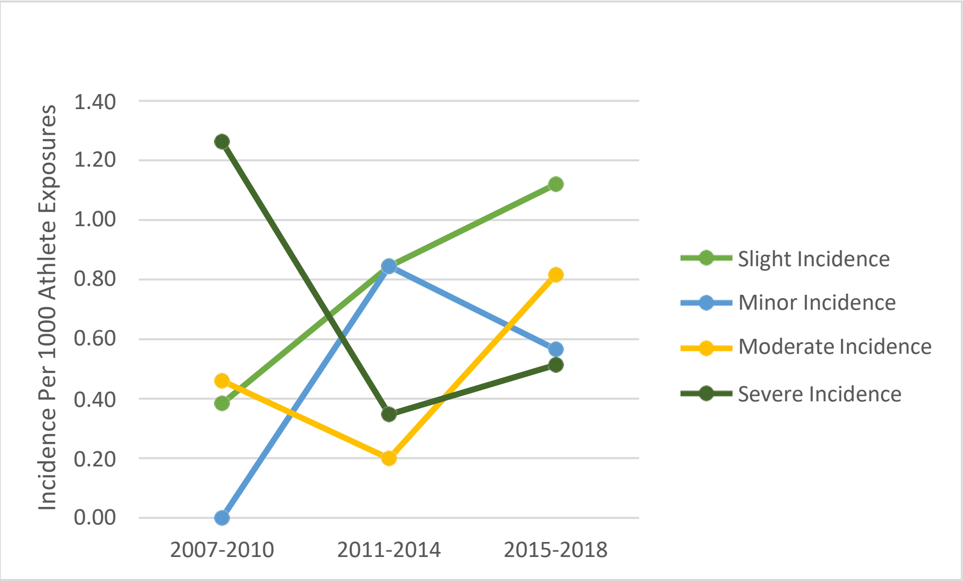

© 2021 Martin CL et al. *JAMA Network Open.*

eFigure 15. 4-Year Groin/Hip/Thigh Time Loss of Veteran Players

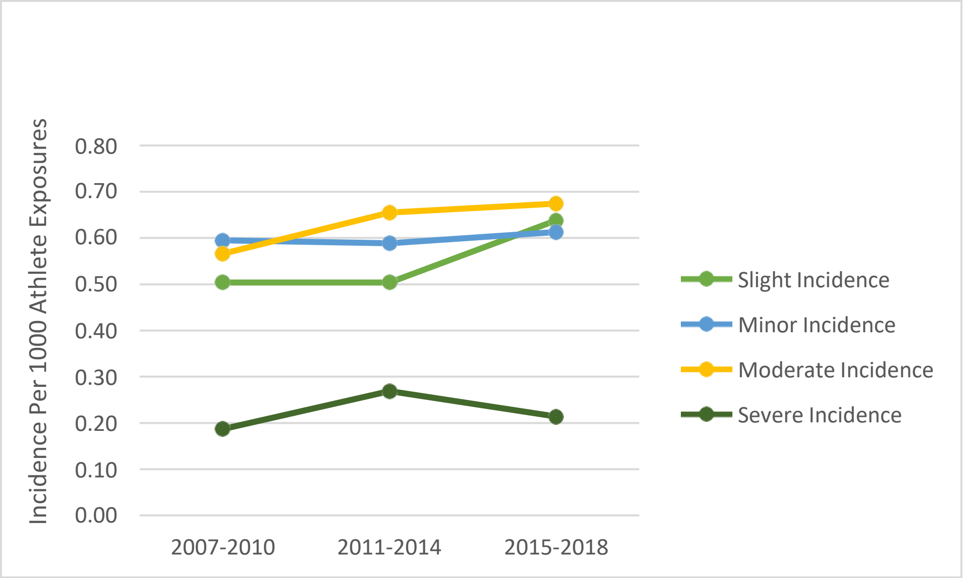

© 2021 Martin CL et al. *JAMA Network Open.*

eFigure 16. 4-Year Groin/Hip/Thigh Time Loss of Rookie Players

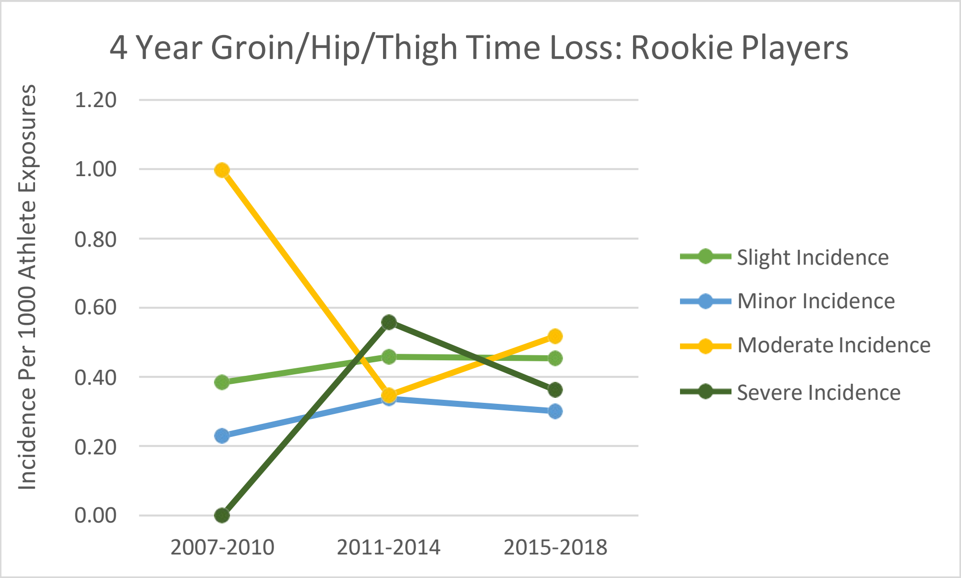

© 2021 Martin CL et al. *JAMA Network Open.*

eFigure 17. 4-Year Concussion Time Loss of Veteran Players

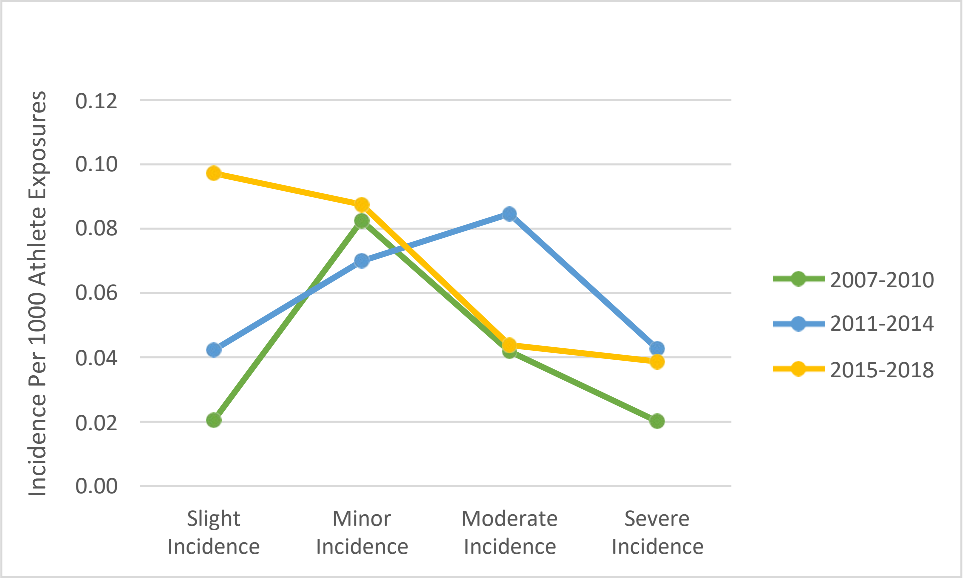

© 2021 Martin CL et al. *JAMA Network Open.*

eFigure 18. 4-Year Concussion Time Loss of Rookie Players

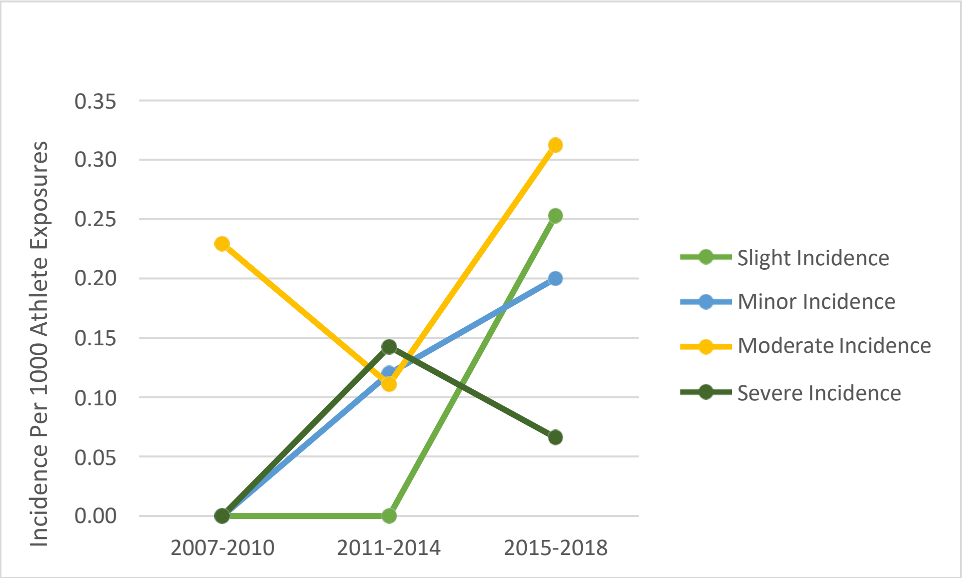

© 2021 Martin CL et al. *JAMA Network Open.*

eTable 5. Mean Difference of Injury Status by Location, Severity, and Rookie Year

**Season Mean Incidence for Time Loss Injury**					
	**Overall**	**Slight Incidence**	**Minor Incidence**	**Moderate Incidence**	**Severe Incidence**
Overall	15.76	4.49	3.60	3.67	2.14
Ankle	2.47	0.78	0.70	0.73	0.26
Concussion	0.25	0.06	0.09	0.07	0.04
Knee	2.50	0.71	0.62	0.60	0.59
Groin/Hip/Thigh	1.97	0.54	0.57	0.62	0.23

**Season Mean Incidence for Time Loss Injury (Veteran Players)**					
	**Overall**	**Slight Incidence**	**Minor Incidence**	**Moderate Incidence**	**Severe Incidence**
Overall	13.84	4.52	3.62	3.64	2.07
Ankle	2.40	0.78	0.66	0.72	0.24
Concussion	0.22	0.06	0.08	0.06	0.03
Knee	2.51	0.70	0.63	0.61	0.59
Groin/Hip/Thigh	2.00	0.55	0.60	0.64	0.23

**Season Average Incidence for Time Loss Injury (Injury Rookie Season)**					
	**Overall**	**Slight Incidence**	**Minor Incidence**	**Moderate Incidence**	**Severe Incidence**
Overall	14.88	4.21	3.40	4.15	3.11
Ankle	3.17	0.81	1.08	0.84	0.44
Concussion	0.48	0.12	0.15	0.23	0.06
Knee	2.45	0.81	0.49	0.55	0.55
Groin/Hip/Thigh	1.65	0.44	0.32	0.49	0.32
**4 year Mean Incidence for Time Loss Injury**					
	**Overall**	**Slight Incidence**	**Minor Incidence**	**Moderate Incidence**	**Severe Incidence**
Overall	15.76	4.49	3.60	3.67	2.14
Ankle	2.47	0.78	0.70	0.73	0.26
Concussion	0.25	0.06	0.09	0.07	0.04
Knee	2.50	0.71	0.62	0.60	0.59
Groin/Hip/Thigh	1.97	0.54	0.57	0.62	0.23

**4 Year Mean Incidence for Time Loss Injury (Veteran Players)**					
	**Overall**	**Slight Incidence**	**Minor Incidence**	**Moderate Incidence**	**Severe Incidence**
Overall	13.84	4.52	3.62	3.64	2.07
Ankle	2.40	0.78	0.66	0.72	0.24
Concussion	0.22	0.06	0.08	0.06	0.03

© 2021 Martin CL et al. *JAMA Network Open.*

4 Year Mean Incidence for Time Loss Injury (Veteran Players) Continued					
	**Overall**	**Slight Incidence**	**Minor Incidence**	**Moderate Incidence**	**Severe Incidence**
Knee	2.51	0.70	0.63	0.61	0.59
Groin/Hip/Thigh	2.00	0.55	0.60	0.64	0.23

4 Year Mean Incidence for Time Loss Injury (Injury Rookie Season)					
	**Overall**	**Slight Incidence**	**Minor Incidence**	**Moderate Incidence**	**Severe Incidence**
Overall	14.88	4.21	3.40	4.15	3.11
Ankle	3.17	0.81	1.08	0.84	0.44
Concussion	0.48	0.12	0.15	0.23	0.06
Knee	2.45	0.81	0.49	0.55	0.55
Groin/Hip/Thigh	1.65	0.44	0.32	0.49	0.32

eTable 6. Initial vs Subsequent Injury Incidence by Body Part

Body Part or Illness	Injury Count	Injury Severity (Games Missed)	Injury Incidence (95% CI)
**Overall**	5364	3 (1, 3)	13.82 (13.82, 13.83)
All Injuries	491	4(1, 11)	14.11 (13.60, 14.62)
Initial Injury	4873	3 (1, 9)	13.48 (12.94, 14.01)
Subsequent Injury			
**Ankle**			
All Injuries	957	3 (1, 6)	2.47 (2.31, 2.62)
Initial Injury	712	3 (1,7)	3.42 (3.17, 3.67)
Subsequent Injury	245	2 (1, 6)	1.36 (1.19,1.53)
**Knee**			
All Injuries	974	3 (1, 12)	2.51 (2.35, 2.67)
Initial Injury	462	5 (2, 18)	2.22 (2.02, 2.42)
Subsequent Injury	512	2 (1, 8)	2.84 (2.60, 3.09)
**Groin/Hip/Thigh**			
All Injuries	765	3 (1, 7)	1.97 (1.83, 2.11)
Initial Injury	372	3 (2, 7)	1.79 (1.61, 1.97)
Subsequent Injury	393	3 (1, 6)	2.18 (1.97, 2.40)
**Illness**			
All Injuries	600	1 (1, 3)	1.55 (1.42, 1.67)
Initial Illness	244	2 (1, 4)	1.17 (1.03, 1.32)
Subsequent Illness	356	1 (1, 2)	1.98 (1.77, 2.18)
**Trunk/Back/Buttock**			
All Injuries	566	2 (1, 5)	1.46 (1.34, 1.57)
Initial Injury	344	2 (1, 5)	1.65 (1.48, 1.82)
Subsequent Injury	222	2 (1, 5)	1.23 (1.07, 1.39)
**Foot/Toe**			
All Injuries	385	3 (1, 10)	0.99 (0.89, 1.09)
Initial Injury	191	5 (2, 16)	0.92 (0.79, 1.04)
Subsequent Injury	194	3 (1, 7)	1.08 (0.93, 1.22)
**Forearm/Wrist/Hand**			
All Injuries	324	5 (2, 15)	0.84 (0.74, 0.93)
Initial Injury	161	9 (2, 19)	0.77 (0.65, 0.89)
Subsequent Injury	163	3 (1, 12)	0.91 (0.77, 1.04)
**Shoulder/Arm/Elbow**			
All Injuries	318	3 (1, 9)	0.82 (0.73, 0.91)
Initial Injury	143	5 (1, 13)	0.69 (0.57, 0.80)
Subsequent Injury	175	2 (1, 7)	0.97 (0.83, 1.12)
**Lower leg/Achilles tendon**			
All Injuries	263	4 (2, 10)	0.68 (0.60, 0.76)
Initial Injury	185	4 (2, 11)	0.89 (0.76, 1.02)
Subsequent Injury	78	4 (2, 9)	0.43 (0.34, 0.53)

© 2021 Martin CL et al. *JAMA Network Open.*

**Head/Neck**			
All Injuries	113	2 (1, 5)	0.29 (0.24, 0.34)
Initial Injury	56	2 (1, 5)	0.27 (0.20, 0.34)
Subsequent Injury	57	2 (1, 5)	0.32 (0.23, 0.40)
**Concussion**			
All Injuries	99	3 (2, 6)	0.26 (0.20, 0.31)
Initial Injury	67	3 (1, 5)	0.32 (0.24, 0.40)
Subsequent Injury	32	4.5 (2, 11.25)	0.18 (0.12, 0.89)
Slight Injury = 1 game missed; Minor Injury = 2-3 games missed; Moderate Injury = 4-13 games missed;<br>Severe Injury = 14+ games missed			
Incidence were compared between players who did or did not sustain an injury during their rookie season			

© 2021 Martin CL et al. *JAMA Network Open.*

eTable 7. Poisson Regression Results

Coefficients	Beta	SE	Z value	p value	95% CI
Injury Status Rookie Season	-0.15	0.15	-0.99	0.321	-0.44,0.16
Age	-0.04	0.01	-3.08	0.002	-0.06,-0.014
BMI	0.04	0.01	3.58	<0.001	0.017, 0.059
Position (Forward)	-0.04	0.05	-0.81	0.416	-0.136, 0.057
Position (Guard)	0.02	0.05	0.34	0.732	-0.082, 0.117
Rookie (Yes or No)	-0.12	0.005	-22.18	<0.000	-0.131, -0.109
Draft Pick	-0.01	0.001	-3.93	<0.000	0.007, -0.002
Injury Severity (Minor)	0.2	0.16	1.3	0.193	-0.116, 0.494
Injury Severity (Moderate)	0.21	0.16	1.34	0.18	-0.111, 0.506
Injury Severity (Severe)	0.12	0.16	0.65	0.515	-0.227, 0.417
Injury Severity (Slight)	0.31	0.16	1.88	0.06	-0.026, 0.618
SE=Standard Error; 95%CI= Confidence Interval					

© 2021 Martin CL et al. *JAMA Network Open.*

eTable 8. Rate Ratios for Initial vs Subsequent Injuries in Rookie vs Veteran Athletes

	Rookie		Veterans	
Body Part or Illness	Rate Ratio	95% CI	Rate Ratio	95% CI
**Overall**				
Initial Injury	1.41	1.29, 1.53	0.71	0.59, 0.83
Subsequent Injury	1.01	0.10, 1.21	0.99	0.79, 1.18
**Ankle**				
Initial Injury	1.41	1.19, 1.64	0.71	0.48, 0.93
Subsequent Injury	1.06	0.63, 1.49	0.94	0.51, 1.37
**Knee**				
Initial Injury	1.00	0.68, 1.37	1.00	0.68, 1.32
Subsequent Injury	0.92	0.60, 1.23	1.09	0.77, 1.41
**Groin/Hip/Thigh**				
Initial Illness	0.94	0.57, 1.31	1.07	0.70, 1.43
Subsequent Illness	0.64	0.22, 1.06	1.57	1.15, 1.99
**Illness**				
Initial Injury	1.55	1.18, 1.92	0.65	0.27, 1.02
Subsequent Injury	0.87	0.49, 1.26	1.15	0.76, 1.53
**Trunk/Back/Buttock**				
Initial Injury	0.67	0.23, 1.11	1.49	1.05, 1.93
Subsequent Injury	0.69	0.15, 1.24	1.44	0.90, 1.98
**Foot/Toe**				
Initial Injury	1.62	1.20, 2.03	0.62	0.20, 1.03
Subsequent Injury	1.04	0.54, 1.54	0.96	0.46, 1.46
**Forearm/Wrist/Hand**				
Initial Injury	1.07	0.54, 1.60	0.93	0.40, 1.46
Subsequent Injury	0.81	0.23, 1.40	1.23	0.64, 1.82
**Shoulder/Arm/Elbow**				
Initial Injury	1.97	1.52, 2.42	0.51	0.06, 0.95
Subsequent Injury	1.04	0.63, 1.46	0.96	0.54, 1.37
**Lower leg/Achilles tendon**				
Initial Injury	0.52	-0.15, 1.19	1.92	1.25, 2.59
Subsequent Injury	0.14	-1.84-2.11	7.33	5.36, 9.31
**Head/Neck**				

© 2021 Martin CL et al. *JAMA Network Open.*

Initial Injury	1.43	0.64-2.22	0.70	-0.09, 1.49
Subsequent Injury	1.00	0.08, 1.92	1.00	0.08, 1.92
**Concussion**				
Initial Injury	2.73	2.14, 3.32	0.37	-0.22, 0.95
Subsequent Injury	8.38	7.82, 8.93	0.12	-0.44, 0.68

Rate Ratio for Initial Injury represents initial incidence rate (IR) for Rookie Players/Initial IR for Veteran Player stratified by body part or illness; Rate Ratio for Subsequent Injury represents incidence rate (IR) for Rookie Players/Initial IR for Veteran Player stratified by body part or illness.

© 2021 Martin CL et al. *JAMA Network Open.*
